# Sirtuins as Interesting Players in the Course of HIV Infection and Comorbidities

**DOI:** 10.3390/cells10102739

**Published:** 2021-10-13

**Authors:** Karolina Jurkowska, Beata Szymańska, Brygida Knysz, Amadeusz Kuźniarski, Agnieszka Piwowar

**Affiliations:** 1 Department of Toxicology, Faculty of Pharmacy, Wrocław Medical University, 50-556 Wrocław, Poland; beata.szymanska@umed.wroc.pl (B.S.); agnieszka.piwowar@umed.wroc.pl (A.P.); 2 Department of Infectious Diseases, Liver Diseases and Acquired Immune Deficiencies, Faculty of Medicine, Wrocław Medical University, 51-149 Wrocław, Poland; brygida.knysz@umed.wroc.pl; 3 Department of Dental Prosthodontics, Faculty of Dentistry, Wrocław Medical University, 50-425 Wrocław, Poland; amadeusz.kuzniarski@umed.wroc.pl

**Keywords:** HIV, sirtuins, HAART, cART, comorbidities

## Abstract

The sirtuins (SIRTs) are a family of enzymes from the group of NAD^+^-dependent deacetylases. Through the reaction of splitting the acetyl group of various transcription factors and histones they regulate many processes in the organism. The activity of sirtuins is linked to metabolic control, oxidative stress, inflammation and apoptosis, and they also affect the course of viral infections. For this reason, they may participate in the pathogenesis and development of many diseases, but little is known about their role in the course of human immunodeficiency virus (HIV) infection, which is the subject of this review. In the course of HIV infection, comorbidities such as: neurodegenerative disorders, obesity, insulin resistance and diabetes, lipid disorders and cardiovascular diseases, renal and bone diseases developed more frequently and faster compared to the general population. The role of sirtuins in the development of accompanying diseases in the course of HIV infection may also be interesting. There is still a lack of detailed information on this subject. The role of sirtuins, especially SIRT1, SIRT3, SIRT6, are indicated to be of great importance in the course of HIV infection and the development of the abovementioned comorbidities.

## 1. Introduction

The number of people infected with HIV and developed acquired immunodeficiency syndrome (AIDS) is still large, but thanks to the introduction of highly active antiretroviral therapy (HAART), the life expectancy of infected people has significantly increased. The incidence of AIDS-defining diseases has decreased, but the development of accompanying diseases is becoming the most important health and social problem among HIV-infected patients [1]. Cohort analysis conducted in the years 1999–2004, among patients in the USA living with HIV (*n* = 68.669), showed a significant increase in mortality (32.8%) due to non-AIDS-related comorbidities (NARCs) [2]. Morbidities, such as: diabetes, cardiovascular diseases, hypertension, hyperlipidemia, kidney diseases, osteoporosis, hepatitis C virus infection, occur much more often in infected people due to accelerated aging processes in HIV-positive cohorts [3,4]. A multicenter, cross-sectional study conducted by Serrão et al. [3] among HIV-infected patients over the age of 50 (*n* = 401) has shown that there is a positive relationship between the age of the patients and the occurrence of NARCs, which include: diabetes mellitus, hypercholesterolemia, arterial hypertension, acute myocardial infarction, stroke, renal failure, renal lithiasis, chronic hepatitis C (CHC), chronic hepatitis B, emphysema/bronchitis, osteoporosis and depression/chronic anxiety (*p* < 0.001), as well as between NARC and duration of infection (*p*< 0.001). It is also noteworthy that nearly 90% of patients had at least one NARC, and 35% had three or more. The most common were: hypercholesterolemia, hypertension, depression/chronic anxiety, chronic hepatitis C and diabetes mellitus, which occurred in over 50% of patients [3].

The selection of appropriate medication and optimization of therapy, also in terms of additionally used drugs, seems to be particularly important to avoid the risk of interaction or lowering the effectiveness of antiretroviral therapy. However, there is still little literature data on the detailed mechanisms of the increased risk of metabolic diseases or accelerated aging [1]. Research on signaling pathways that are involved in the abovementioned disorders is still needed to better understand the pathogenesis of the diseases. The latest literature data indicate that proteins from the sirtuin family play a role in the deepening of these comorbidities or increasing their occurrence in HIV-infected patients.

The aim of this review is to indicate the potential role for sirtuins in the course of HIV infection, as well as their involvement in the development of the abovementioned comorbidities and worsening of HIV patients’ condition. In this review we describe our current understanding of the biological function of the sirtuin (SIRT 1–7) family in the course of HIV infection as well as the development of other diseases—comorbidities not directly induced by HIV infection. This research reviews the literature based on the MEDLINE database using the keywords: sirtuins, HIV, cardiovascular disease, insulin resistance, diabetes, osteoporosis, kidney diseases, neurodegenerative diseases in the years 2009–2020; 3209 records were found, of which 175 were selected for this review.

## 2. Sirtuins Family—The General Role in Organisms

Sirtuins (SIRTs) were first isolated from yeast Sacharomyces cerviesiae. They belong to distinct class III NAD^+^-dependent histone deacetylases (HDACs). In humans, there are seven genes for sirtuins, where SIRT 1 belong to class Ia, SIRT 2 and SIRT3 3 to the class Ib, SIRT 4 to class II, SIRT 5 to class III and SIRT 6, 7 to class IV. This classification was created based on sequence homology to all proteins from the Sir2 (silent information regulator) protein family [5]. Their main function is the reaction of splitting the acetyl group from proteins, transcription factors, histones, etc., with cofactor NAD^+^, and thus the covalent modification of target proteins. Equally importantly, though this is yet to be fully understood, in addition to their deacetylase activity, these proteins can have alternative enzymatic activities, including ADP ribosylation (SIRT1, SIRT4 and SIRT6), desuccinylation and demalonylation (SIRT5), delipoylation (SIRT4) and depalmitoylation and demyristoylation (SIRT6). This breadth of functions is partly derived from sirtuin’s diverse intracellular localizations: nuclear (SIRT1, SIRT6 and SIRT7), cytoplasmic (SIRT2), mitochondrial (SIRT3, SIRT4, SIRT5) [6,7,8,9]. The enzymes of the sirtuin family have similar chemical structure in both prokaryotes and eukaryotes. Characteristic elements of their structure are: (1) a catalytic domain consisting of about 260 amino acid residues, (2) two key subdomains: Rossman’s NAD^+^ binding folder and zinc ions binding subdomain, (3) an enzyme active site formed between subdomains, (4) a NAD^+^ binding pocket [10].

Detailed information about their localization, function, role and main molecular targets are well described in many scientific publications [6,7,8,9], so therefore, we will not describe this data in detail in this study, but we will only present the most important information in terms of potential grab points for the action of sirtuins, which are common in the pathomechanism of HIV infection and the development of accompanying diseases.

## 3. Role of Sirtuins during HIV and Other Viral Infections

Given the diverse localizations and activities described above, sirtuins are core regulators of metabolism and transcription. They have the ability to control numerous cellular pathways required throughout the viral life cycle, but the exact role of sirtuins in viral infections has not yet been fully understood [11].

### 3.1. Viral Targets and Sirtuin Action

Sirtuins can potentially affect viral factors and influence the course of infection. An example of this type of interaction is the deacetylation of the Tat HIV viral protein by SIRT1, which affects the efficiency of transcription of the integrated viral genetic material [12]. Viral Tat protein influences chromatin modifying factors in a manner that is favorable to viral replication, including: recruiting histone acetyltransferase 1, increasing the level of Tat acetylation and increasing the efficiency of the RNA transcription process, contributing to an increase in viral load [13,14]. Deacetylation of Tat by SIRT1 causes its reconstitution and recruitment of appropriate host proteins, including cyclin T1 to TAR element at the 5’ end of viral RNA, preventing the termination of the transcription process at the elongation stage and starting the next transcription cycle. Without Tat protein, the elongation process is ineffective, resulting in significantly higher levels of interrupted and fewer full-length viral transcripts [15]. Tat is also a factor directly affecting SIRT1, binding to its catalytic domain and thereby blocking deacetylase activity relative to NF-kB, p53, p21 or BCL2-associated X protein (BAX), causing an HIV-specific status of chronic immune activation [16]. By inducing a state of chronic immune activation due to HIV infection, the production of transcription factors and pro-inflammatory interleukins, among others: NF-kB and IL-2, is intensified, which allows the integration of the newly formed viral DNA into the host genome [17,18].

The greatest number of studies on the relationship between SIRT1 and HIV Tat protein concern IL-2 and NF-kB, the activity of which is crucial in the course of infection. Activation of IL-2 gene expression occurs via the T cell receptor (TCR) and the CD28 coreceptor. The virus protein Tat is involved in the process activation of the IL-2 gene and regulates expression of host genes in infected T cells [17]. As a result, the activation of T cells via TCR receptors causes cleavage of the NF- kB from Inhibitor of NF- kB (IkB) inhibitory factor, its translocation to the nucleus and activation due to post-translational modifications, including lysine acetylation by SIRT1 [19]. In vitro studies have shown a reduction in NFkB-p65, a subunit deacetylation in the presence of Tat as well as in the presence of nicotinamide (NAM)-factor, limiting the activity of SIRT1. Jurkat T cells vectored with Tat showed a 1200-fold increase in IL-2 mRNA levels in Tat infected cells, compared with only 250-fold increase in control cells. Mouse SIRT1^−/−^ and SIRT1^+/+^ embryonic fibroblasts exposed to tumor necrosis factor α (TNFα) to activate NF-kB showed an increased copy number of mRNA IκBα and NF-kB responsive product in SIRT1^−/−^ mice. The introduction of Tat protein increased expression of IκBα in SIRT1^+/+^ mice (5–11 times) and in SIRT1^−/−^ mice (2–3 times) [15].

Tat also negatively affects SIRT1 activity by affecting the NAD^+^/NADH ratio, a key factor for the modulation of its activity. Zhang et al. [20] showed on Hela-CD4-β-gal (MAGI) cell lines that the Tat protein inhibited nicotinamide phosphoribosyltransferase (NAMPt), an enzyme converting NADH to NAD^+^ [20]. The exposure of MAGI cells to resveratrol increased NAD^+^ levels and SIRT1 expression. Exposure to the SIRT1 inhibitor NAM and silencing of the SIRT1 gene by siRNA enhanced the activating effect of Tat on HIV transcription [12].

### 3.2. Host Targets and Sirtuins Action in Viral Infections

In vitro studies have shown that silencing the SIRT1 gene in MRC5 cells by small interfering RNA (siRNA) leads to increased replication efficiency of viruses, such as: herpes simplex virus-1 (HSV-1), influenza virus H1N, human cytomegalovirus (HCMV), adenovirus type 5 (Ad5), whereas SIRT1 activators, e.g. resveratrol (RSV) or CAY10602 (more selective for SIRT1 synthetic agent) reduced viral replication efficiency [11,13]. Such a broad antiviral spectrum of SIRT1 may be associated with metabolic activity and influence a number of host processes, such as glycolysis, fatty acid synthesis and fatty acid oxidation. SIRT1 action can prevent changes in cell metabolism that accompany some viral infections, including: HSV-1, HCM, HCMV, and keep metabolic homeostasis [13]. 

SIRT1 can affect viral replication through its effect on host transcription factors, which can be used in the course of a viral infection. These include: nuclear factor kappa-light-chain-enhancer of activated B cells (NF-κB), forkhead box protein O1 (FOXO1) or p53 [13]. Bogoi et al. [14] showed that people infected with HIV had a significantly higher gene expression of SIRT1 in isolated CD4+ cells, compared to the control group of non-infected subjects. Expression of genes encoding chromatin-modifying enzymes such as: methyltransferases, acetylases and deacetylases, including SIRT1, are altered in HIV-infected individuals, in order to create an appropriate environment for virus replication and the progression of infection [14].

**Figure 1:**
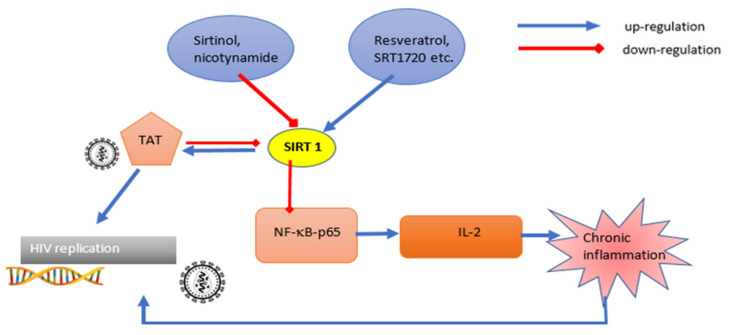
Scheme of interaction of SIRT1 1 with viral protein Tat [12]. Deacetylation of Tat by SIRT1 causes its reconstitution, preventing the termination of the transcription process at the elongation stage, starting the next HIV transcription cycle. Tat also directly affects SIRT1 by binding to its catalytic domain and thereby blocking deacetylase activity relative to NF-kB, causing production of transcription factors and pro-inflammatory interleukins, among others: IL-2, and as a result, HIV-specific status of chronic immune activation, which allows to integrate the newly formed viral DNA into the host genome. SIRT 1, sirtuin1; IL-2, interleukin 2; Tat, trans-sctivator of transcription; NF-κB, nuclear factor kappa-light-chain-enhancer of activated B cells.

SIRT 1 is believed to be one of the key factors in maintaining the anergy state of T cells. Up-regulation of SIRT1 causes a reduction in the expression of induction factors of T cells: NF-kB, activator protein 1 (AP-1) and IL-2 [14,16]. Gao et al. [16] showed on naive CD4+ lymphocytes isolated from transgenic mice that lymphocytes showed a potential mechanism of switching to an anergic state through the regulation of SIRT1 expression. The authors noted that the highest expression of SIRT1 occurs in anergic (latent) T cells, compared to activated, naive and differentiated effector T cells. T cells become anergic by stimulation of the TCR receptor without costimulation of the CD28 receptor. This process is a natural mechanism in the prevention of autoimmune processes. After TCR stimulation by anti CD28 and anti CD3, a significant increase in SIRT1 expression was observed. The authors showed that FOXO3a binds to the promoter for SIRT1 via EGR2 and EGR3, activating SIRT1 transcription peripheral T cell tolerance. By the reverse process of activating T cells, IL-2 sequesters FOXO3a into the cytoplasm, preventing the enhanced expression of SIRT1, which causes reversion of T cell anergy [16]. In ex vivo studies using CD8-depleted mononuclear cells (PBMCs) isolated from peripheral blood of HIV-infected patients treated with antiretroviral drugs, without detectable viremia, Samer et al. [17] demonstrated the ability of sirtuins inhibitor NAM to reverse HIV-1 latency by inhibiting histone deacetylation and inhibiting chromatin condensation. As a result, the latency was reversed and HIV was actively expressed. NAM has been shown to be more effective in reversing HIV latency and achieving serological remission compared to the combination of methyltansferase inhibitors (MTI): chaetocin and BIX01294 [17]. The described results may indicate the potential activity of SIRT1 as a latency reversing agent, which may be an interesting area for further research. Figure 1 shows a scheme of the effect of SIRT1 on the viral Tat protein and HIV transcription.

## 4. Liver Disturbances in HIV and Sirtuin’s Role

Literature data show that HIV patients are a group at risk of liver damage, hepatic inflammation and fibrosis development resulting from HIV infection itself, as well as the use of HAART [21]. In a Swiss cohort study (*n* = 2365) conducted between 2002–2008, 16% of participants developed an elevated alanine aminotransferase (ALT) level during the follow-up period, which was associated with HIV RNA above 100,000 copies/mL, Stavudine (d4T) and Zidovudine (AZT) treatment [22]. On the other hand, effective treatment significantly reduces the risk of severe liver diseases in HIV-infected patients [23]. The coreceptors of HIV: CC chemokine receptor 5 (CCR5) and C-X-C chemokine receptor type 4 (CXCR4), are present in hepatocytes, activated hepatic stellate cells (HSCs) and other liver cells, which explains the direct effect of the virus on the liver. Moreover, the presence of HIV RNA, proviral DNA and viral proteins has been demonstrated in all liver tissues [18,24]. Additionally, an almost twofold increase in the synthesis of collagen I, characteristic of fibrosis, and an 80-fold increase in MCP-1 levels in primary HSCs 48 h after HIV infection were shown, which indicate a direct pro-inflammatory and profibrogenic viral effect on HSCs. The profibrogenic effect in HSCs of the viral gp120 was also demonstrated by Bruno et al. [22], who observed increased expression of IL-6, chemoattractant protein-1 (MCP-1) and tissue inhibitor of metalloproteinase-1 (TIMP-1), which promotes liver fibrosis [22].

Among HIV-positive patients, hepatitis B virus (HBV) and hepatitis C virus (HCV) co-infection are common, which significantly increases the risk of liver cirrhosis and fibrosis. It is estimated that HCV co-infection occurs in approximately 5.8% of HIV-infected people [25]. Liver fibrosis is a reversible condition, caused by the activation of HSCs by platelet-derived growth factor (PDGF) or transforming growth factor β (TGF-β). As a result, HSCs increase the synthesis of collagen and extracellular matrix, which are the cause of liver fibrosis. HIV infection, even if successfully treated, accelerates HCV-induced liver fibrosis and leads to the earlier development of end-stage liver disease (ESLD) [26].

The influence of SIRT1 action in the development of CHC has been demonstrated. Li et al. [25] showed a significantly lower SIRT1 expression and increased levels of acetylated p53 in the liver tissues of CHC patients compared to the healthy controls. The decrease in SIRT1 expression was correlated with increased serum levels miR-34a and ac-p53, which suggests that SIRT1 may be inhibited by miR-34a, promoting apoptotic processes in hepatocytes [25].

Gupta et al. [26] noticed increased expression of profibrogenic factors, such as: TGF-β, matrix metalloproteinase-2 (MMP-2) and IL-6 in human hepatic stellate cells (LX-2) exposed to supernatants from HIV-infected PBMCs, compared to LX-2 treated uninfected PBMC supernatants. The authors also showed that LX-2 changed the expression of over 60 miRNAs, including those related to TGF-β signaling and the regulation of the expression of the profibrogenic genes: collagen type I alpha 1 chain (Col1a1), collagen type I alpha 2 chain (Col1a2), and collagen type III alpha 1 chain (Col3a1) and mothers against decapentaplegic homolog (SMADs) [26]. Research shows that the expression of the abovementioned genes and the activity of TGF-β is also regulated by SIRT6, which indicates its potential role in the development of hepatic disorders in HIV-infected people. The authors also showed that as a result of exposure to supernatants of HIV-infected PBMCs in the tested LX-2 cells, increased NF-κB p65 phosphorylation was observed, promoting pro-inflammatory and profibrotic effects [27].

Zhang et al. [27] showed that expression of SIRT6 was significantly reduced in HSCs isolated from mice fibrotic livers. Specific SIRT6 knockout in mouse primary HSCs resulted in activation of HSCs in a TGF-β dependent manner. Increased mRNA expression of fibrogenic genes: alpha-smooth muscle actin (α-SMA), Col1a1, Col1a2 and collagen type III alpha 1 chain Col3a1 was also noted. In contrast, the selective agonist of SIRT6- ML-800 inhibited the expression of the abovementioned genes, and the TGF-β-induced fibrosis in LX-2 cells. SIRT6 has also been shown to affect TGF-β in HEK293 cells by deacetylating lysine 54 of mothers against decapentaplegic homolog (SMAD) 2- profibrogenic transcription factor, inhibiting its cytosol to nucleus translocation induced by TGF-β and its recruitment to the promoters of fibrogenesis genes; Col1a1 and Col1a2 acting protectively against liver fibrosis [27]. As shown by the above data, SIRT6, through down-regulation of TGF-β signaling and activation of related profibrogenic genes, indicates the participation of SIRT6 in the pathogenesis of liver fibrosis development in the course of HIV infection, eliminating the profibrotic effect of the virus.

It is mentioned that mitochondrial SIRT3 also has anti-fibrotic and anti-inflammatory activity. It deacetylates glycogen synthase kinase 3β (GSK-3β), increasing its activity, which in turn weakens TGF-β signaling in a mechanism dependent on β-catenin and SMADs. SIRT3 also contributes to the reduction in lipid accumulation in hepatocytes by activating AMPK signaling, weakening NF-κB inflammatory activity [28,29,30]. Li et al. [29] have shown that SIRT3 in NAFLD protects hepatocyte function by inhibiting OS and mitochondrial apoptosis via the ERK-CREB signaling pathway [29]. SIRT3 activation also increases the activities of antioxidant enzymes, such as CAT, SOD and GPx, showing a prevalent effect on liver fibrosis by reducing oxidative stress (OS) [31,32]. Increased oxidative stress is considered to be one of the causes of liver damage in HIV patients [31].

The literature data indicate the role of sirtuins as important regulators of hepatic metabolism, associated with, among others, carbohydrate and energy management. The effect of sirtuins on carbohydrate metabolism has been described earlier (point six). Calorie restriction increases NAD^+^/NADH ratio, which activate SIRT1, resulting in activation of PPARα-, the main transcription factor regulating lipid metabolism during feeding and fasting periods. During fasting it intensifies beta oxidation, while during feeding causes de novo synthesis of lipids in the form of triglycerides, which are the reserve material in the liver [31]. In mice with NAFLD induced by a high fat diet, SIRT1 is a factor regulating the activity of QKI5-RNA-binding proteins—signal transduction and activation of RNA (STAR proteins)—which affect, among others, the synthesis of triglycerides in the liver. The exact function of QKI5 in the liver is not yet known. Western blot analysis and immunohistochemistry examination conducted by Zhang et al. [32] showed decreased liver expression of SIRT1, QKI5, FOXO1 and PPARα in the tested NAFLD mice when compared to the control group. Increased QKI5 acetylation in hepatocytes of the tested animals after the silencing of gene expression for SIRT1 with siRNA or after nicotinamide treatment was noted. An increase in FOXO1 and PPARα was also observed in SIRT1 agonist SRT1720-treated hepatocytes, and an inverse relationship has been demonstrated after exposure of hepatocytes to nicotinamide. It was also shown that the level of TG in hepatocytes was inversely proportional to the activity of SIRT1, significantly up-regulated by SRT170 and down-regulated by nicotinamide. The authors suggested that SIRT1 may be considered as a factor that regulates lipogenesis in hepatocytes by QKI5 via the FOXO1/PPARα signaling pathway. This was confirmed by studying the effect of the FOXO1 inhibitor AS1842856, which reduced FOXO1 and PPAR expression without affecting SIRT1 and QKI5. This effect was reversed under the influence of SRT1720 [32]. Studies on human fetal hepatocytes have shown that inhibition of SIRT1 increased de novo lipogenesis. Significantly increased expression of genes responsible for the synthesis of fatty acids was noted: acetyl-CoA carboxylase (ACC), stearoyl-CoA desaturase (SCD) and elongase of long chain fatty acids family 6 (ELOVL6) after exposure to the SIRT1 inhibitor, sirtinol (50 uM), compared to control [33].

**Figure 2:**
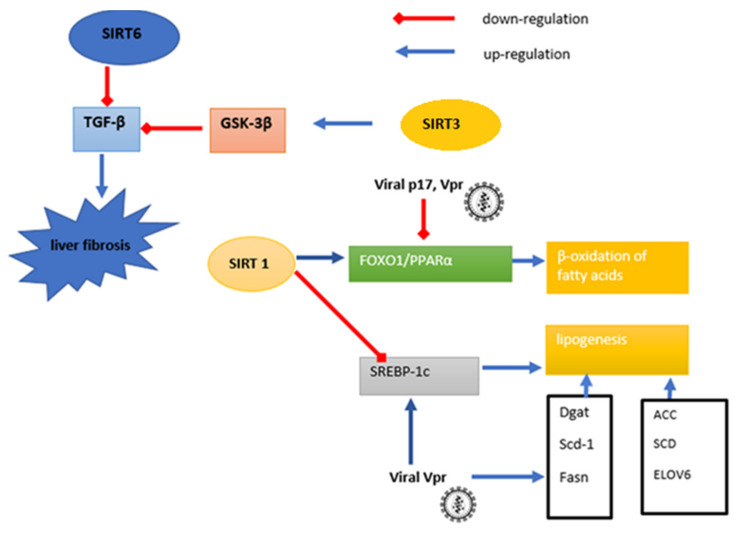
Scheme of connection between sirtuins, HIV and hepatic metabolism. SIRT3 deacetylates GSK-3β, increasing its activity, which in turn weakens TGF-β signaling. SIRT6, through down-regulation of TGF-β signaling reduces the expression of profibrogenic genes. SIRT1 up-regulates fatty acid oxidation in hepatocytes via the FOXO1/PPARα signaling pathway. Viral protein Vpr acts opposite to SIRT1 to increase expression of genes responsible for the synthesis of fatty acids by increasing SREBP1c activity. SREBP-1c, sterol regulatory element binding protein-1c; FOXO1, forkhead box protein O1; PPARα, peroxisome proliferator-activated receptor α; TGFβ, transforming growth factor β; GSK-3β, glycogen synthase kinase 3 beta.

As shown, about 30–70% of HIV-positive patients develop NAFLD without the involvement of HCV infection [34,35]. Studies in vpr transgenic mice (Vpr-Tg) showed a statistically significant higher fatty acid synthesis rate compared to WT mice. In addition, beta-oxidation of fatty acids was shown to be much less efficient in Vpr-Tg mice compared to WT mice. In the Vpr-Tg group, reduced expression of PPARα was also noted, compared to controls [36]. Sirtuin 1 regulates lipid metabolism directly via the SREBP-1, a key factor controlling the synthesis of fatty acids and triglycerides. During fasting, SIRT1 stimulates fat oxidation and inhibits their synthesis through SREBP-1c. SIRT1 deacetylates SREBP-1c in Lys-289 and Lys-309. Deacetylation reduces the stability of SREBP-1 and interaction with promoters of lipogenesis enzymes genes. The interaction of SIRT1 and SREBP-1c has been proven both in vitro, in mouse hepatoma cells (Hepa1c1c7) and HepG2 cell model, and in vivo (8–10-week-old male BALB/c mice). An increase in the acetylation level of SREBP-1c in the livers of fasted animals with a silenced gene for SIRT1 (injected with adenoviral siSIRT1) compared to the control group (infected with adenovirus without siRNA for SIRT1) was shown. The SREBP-1c acetylation level was higher after treatment with glucose and insulin (to mimic feeding conditions) in HepG2 cells. After administration of 10 mM of the NAM-SIRT1 inhibitor, SREBP-1 acetylation was also significantly increased compared to control cells. High fat diet-induced obesity mice also showed increased levels of SREBP-1c, reduced hepatic levels of SIRT1, increased expression of the lipogenesis genes: SREBP-1c and Fas, and decreased expression of genes associated with fatty acid oxidation: ECI (3,2-trans enoyl-CoA-(∆) isomerase) and MCAD (medium-chain acyl-coenzyme A dehydrogenase) compared to the control group [37]. Figure 2 shows schematically the participation of Sirtuin 1, Sirtuin 3 and Sirtuin 6 in the pathogenesis of liver disorders during HIV infection.

## 5. Cardiovascular Risk in HIV-Infected Patients and Sirtuin Participation

An increased risk of cardiovascular disease is observed in HIV-positive patients. This is connected with the prevalence of metabolic disturbances and HIV-accompanying diseases: insulin resistance, dyslipidemia or hepatitis C, and changes in the immune system connected with HIV infection [38]. As epidemiological data show, increased mortality from cardiovascular disease in such patients is observed, and it is one of the most common causes of non AIDS-related deaths in HIV-positive patients successfully treated with antiretroviral therapy [39]. According to the latest conducted meta-analysis, covering 32 original papers published in the period 1999–2008, the risk of myocardial infarction (MI) in HIV-positive people is significantly higher compared to the uninfected (relative risk: 1.73). An increased risk of MI has also been demonstrated depending on: CD4+ cell count, plasma viral load and long-term use of HAART, especially protease inhibitors (PIs), lopinavir/ritonavir (LPV/r) or indinavir (IDV) [40]. An increased risk in HIV patients of cardiovascular events, such as coronary heart disease, coronary calcification, silent myocardial ischemia, myocardial infarction and elevation of intima-media thickness has also been shown [41]. In a meta-analysis covering nine studies with 1229 HIV-positive patients and 1029 controls, the increased prevalence of non-calcified coronary plaques (NCP) was shown in HIV-positive patients on an average of 58% vs 17% compared to controls, and the risk of NCP was inversely correlated with CD4+ counts [42].

Excessive reactive oxygen species (ROS) production is also one of the key risk factors for CVD. Excessive free radical (hydroxyl, superoxide, hydrogen peroxide, lipid peroxyl) production causes a reduction in nitric oxide (NO) release as well as disorders in posttranslational modification processes endothelial nitric oxide synthase (eNOS), caveolin-1 and increased production of eNOS inhibitor, asymetric dimethylarginine (ADMA), due to increased enzymatic activity of arginine-1 protein transferase (PRMT1). There are literature data showing the involvement of SIRT1 in the prevention of endothelial disorders as a result of OS [43].

In response to oxidative stress, caveolin-1 is phosphorylated in Tyr-14; this form sequesters SIRT1 to caveolae, reduces its amount in the cytosol and causes loss of its deacetylation capacity. Therefore, caveoline-1 can be considered as a potential inhibitor of SIRT1, by the binding of the caveolin-1 scaffolding domain (CSD) to the caveolin-binding domain (CBD) of SIRT1, leading to reduced NO production, and, as a result, endothelial dysfunction [44]. As a result of the absence or decreased SIRT1 activity, hyperacetylated PRMT1 causes a greater level of ADMA-eNOS inhibitor. SIRT1 is also responsible for deacetylation of eNOS at Lys-496 and Lys-506. This process increases enzyme activity and the production of NO, which prevents imbalance between endothelium-derived vasoconstrictors and relaxing factors, considered as a predictor of cardiac events [45]. Endothelial SIRT1 activity against eNOS is promoted by apurinic/apyrmidinic endonuclease 1/redox factor-1 (APE1/Ref-1)—an endonuclease that affects vascular homeostasis. It prevents inactivation of SIRT1 by free radical oxidation, through keeping sulfhydryl groups of cysteine in SIRT1 in a reduced form [46].

The decreased activity of SIRT1 also causes an increase in the amount of acetylated p53 in starting the process of apoptosis in endothelial cells (ECs) [47]. Inhibition of apoptosis in endothelial cells has been shown for viral gp120, which activates caspase 3, Bax and p38 MAP kinase signaling [41]. SIRT1 also deacetylates FOXO1, which has a positive effect on the regulation of apoptosis and cell cycle regulation. The FOXO1/SIRT1 signaling pathway also regulates the redox status of endothelium and maintains vascular homeostasis through an impact on vascular smooth muscle cells. Acetylated FOXO1 reduces the concentration of SIRT1 as well as catalase antioxidant enzymes (CAT) and manganese superoxide dismutase (MnSOD). SIRT1 also deacetylates Lys-81 of the p66Shc adapter protein, inhibiting its transcription by blocking its interaction of acetyl histone 3 with the promoter region for this protein, thereby preventing OS induction by p66Shc through the following mechanisms: down-regulation of GPx, MnSOD and APE1/Ref-1 and activation of membrane-associated NADPH oxidases [48].

Intercellular adhesion molecule 1 (ICAM-1) and vascular cell adhesion molecule 1 (VCAM-1) are also important factors in the development of endothelial dysfunction and atherosclerotic plaque formation, by facilitating the transmission of leukocytes through the epithelium and their adhesion [49]. Jiang et al. [50] demonstrated in human coronary artery endothelial cells (HCAECs) and porcine coronary artery rings treated for 16 h with recombinant gp120 (1 µg/mL) with or without the pretreatment of TNFα (as ICAM-1 inductor) that eNOS expression significantly decreased in both cell types with TNFα pretreatment compared to unexposed cells, and increased the expression of ICAM-1 in both cell types was shown: 5-fold under TNFα and about 80% after exposure to gp120, compared to controls. The use of ICAM-1 siRNA or specifically anti gp120 antibody inhibited conversion to eNOS expression caused by gp120 and TNFα. The authors concluded that TNFα and gp120 act synergistically and induce negative changes in endothelium, which may indicate a new mechanism for inducing endothelial disorder in the course of HIV infection [50]. Liu et al. [51] revealed the reversal of the viral gp120 effect of resveratrol (SIRT1 activator) on human umbilical vein endothelial cells (HUVECs). A dose-dependent (treatment 35, 40, 45 or 50 μM of resveratrol) decrease in ICAM-1 expression was noted. This study also showed that inhibition of ICAM-1 in TNFα treated cells was mediated by the inhibitory effect of resveratrol on the phosphorylation of p65 and IκB, activating NF-κB, thereby inhibiting the inflammatory process. The authors suggest that the effect of resveratrol on ICAM-1 can be mediated via the AMPK/p38/NF-κB signaling pathway [52]. The above data suggest that the mechanism of action of resveratrol is SIRT1 dependent. SIRT1 deacetylates Lys-310 in the RelA/p65 complex in NF-κB, suppressing its pro-inflammatory activity in response to OS, and inhibits the synthesis of pro-inflammatory cytokines or thrombotic factors (ICAM-1, VCAM-1) [52,53]. Pan et al. [53] have shown that resveratrol reverses the inhibitory effect of TNFα on SIRT1 expression. HUVECs treated with resveratrol (0, 5, 10 and 20 μM) showed a significantly decreased expression of SIRT1 after stimulation with 10 μg/L TNFα cells compared to controls. In addition, exposure of HUVECs to SIRT1 siRNA resulted in significantly increased expression of p65 and CD40, which was down-regulated after exposure to resveratrol. Silencing of SIRT1 expression also compensated the inhibitory effect of resveratrol on ROS production, which indicates that the antioxidant and anti-inflammatory effects of resveratrol are mainly related to SIRT1 activation. The authors point to a potential mechanism of SIRT1’s action in protection against OS, by inhibiting TNFα-induced NFkB expression by attenuating p38 MAPK phosphorylation, which in turn promotes the nuclear translocation of p65 [53].

The viral Tat protein, which interacts with SIRT1, as described earlier (point four), also affects the risk of CVD through its effect on EC apoptosis and the development of inflammation. This occurs mainly in an NF-κB-dependent manner, increasing the expression of pro-inflammatory factors: ICAM-1, IL-1β, MCP-1, VCAM-1 and E-selectin, which has been shown on HUVECs and animal models [54]. Tat protein contributes to the apoptosis of ECs through its effect on the secretion of the TNFα and activation of the Fas–FasL-dependent pathway. Tat also induces ROS production through the impact on NF-kB, NADPH oxidase, MnSOD and glutathione GSH levels [55,56]. Endothelial dysfunction is also caused by another viral protein, Nef. Wang et al. [55] showed that Nef is present in the vascular cells, initiating atherosclerotic lesions. The authors showed that the induction of changes in endothelium results from two independent mechanisms: NF-kB and the production of MCP-1, and through the endothelial cell apoptosis via NADPH oxidase-dependent mechanism [55].

It has been shown that SIRT6 can affect the IGF-1/Akt signaling pathway, which regulates systemic aging processes and induces hypertrophy of myocardial cells in the myocardium, increasing predisposition to heart failure by deacetylating histone H3K9 in regions of promoters of IGF-1 up-regulating genes. SIRT6 influences the down-regulation of IGF-1/Akt through the c-Jun transcription factor. Studies in animal models have shown that mice with SIRT6 knockout developed concentric cardiac hypertrophy at about 8–12 weeks of age. Changes in the area of cardiomyocytes have also been noted, including their increased size, mitochondrial regression and vacuolization and interstitial fibrosis. Significantly higher levels of apoptotic proteins have also been demonstrated: caspase 3, Bax, TNF-related apoptosis-inducing ligand (TRAIL), Bcl-2-like protein 1 (Bim), Fas ligand (FasL) and cyclin-dependent kinase inhibitor 1B (CDKN1B) [56].

Some literature data have shown that mitochondrial sirtuins (SIRT 3, 4 and 5) also affect the cardiovascular system. The protective role of SIRT3 is primarily associated with antioxidant protection through the activation of SOD2 and CAT through deacetylation of the FOXO3a and protection against cardiomyocyte apoptosis [57,58]. SIRT3 in endothelial cells regulates the glycolysis process. ECs have the ability to perform metabolic reprogramming (so-called “metabolic flexibility”), which allows them to switch from oxidative phosphorylation to the use of glycolysis as the main source of energy. This mechanism allows the maintainance of tissue homeostasis and protects cells against the increased demand for energy during OS, hypoxia or tissue damage. In cultured ECs derived from SIRT3 knockout mice, damage to the glycolysis and glycolytic capacity process as well as reduced expression of 6-phosphofructo-2-kinase/fructose-2,6-biphosphatase 3 (FKFB3)—a factor that participates in the synthesis of the allosteric activator of PFK-1, fructose 2,6-bisphosphate—was observed. The above data indicate the participation of SIRT3 in the regulation of metabolic flexibility in a PFKFB3-dependent manner, by maintaining high glycolytic activity in the ECs [59].

The role of SIRT4 in CVD is also indicated, but the exact mechanism of action is unknown. Data indicate its high expression in the heart, and it is believed to be a regulating factor for heart hypertrophy in an angiotensin II (Ang-II)-dependent mechanism. Luo et al. [60] showed significantly less Ang-II -induced cell growth as well as decreased production of atrial natriuretic peptide (ANP), inhibiting the renin-angiotensin-aldosterone system (RAS) in cultured SIRT4 knockdown neonatal rat cardiomyocytes (NRCMs). This is a key mechanism in the regulation of not only cardiac hypertrophy but also blood pressure and electrolyte balance, which may indicate an important role of SIRT4 in the pathogenesis of CVD. After chronic Ang-II infusion for 4 weeks, SIRT4 knockout mice showed a significantly lower increase in heart weight to body weight ratio (HW/BW) compared to WT mice (8.9% vs 28.3%), which suggests the key role of SIRT4 in this process. In addition, SIRT4 knockout mice showed significantly greater heart ROS production compared to WT during cardiac hypertrophy, which is an unfavorable factor in the development of cardiac disorders [61]. In contrast to SIRT3, SIRT4 decreases mitochondrial MnSOD activity. SIRT4 overexpression in NRCMs decreased SIRT3-mediated MnSOD deacetylation and decreased its activity, promoting ROS accumulation during hypertrophic stress-inducing cardiovascular disorders [51].

**Figure 3:**
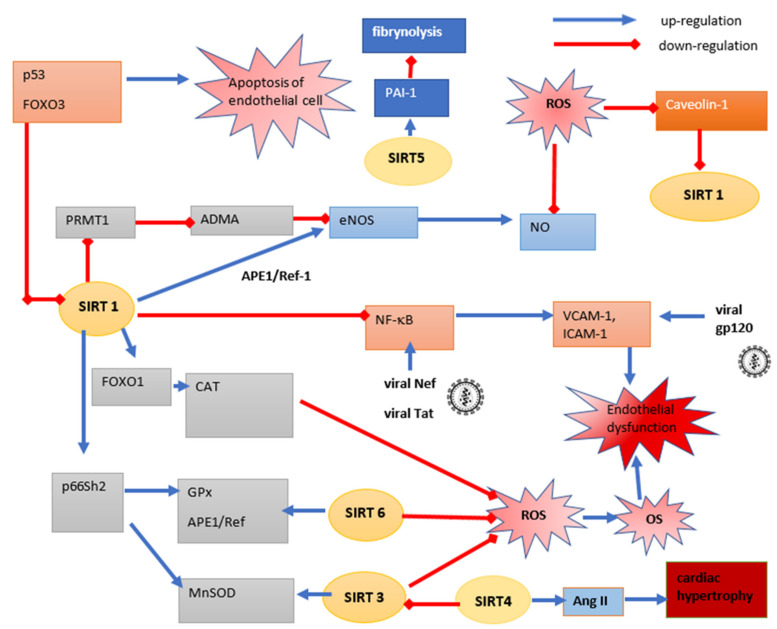
Scheme of connection between cardiovascular disorders, sirtuins and HIV infection. SIRT1 deacetylate PRMT1 and decrease levels of ADMA—eNOS inhibitor. SIRT1 is also responsible for deacetylation of eNOS, which increases enzyme activity and the production of NO. Endothelial SIRT1 activity against eNOS is promoted by APE1/Ref-1. SIRT1 decrease activity of p53, preventing process of apoptosis in endothelial cells. SIRT1 also deacetylates FOXO1, which has a positive effect on the regulation of apoptosis and cell cycle regulation. Decetylated FOXO1 increases the concentration of CAT and MnSOD. SIRT1 deacetylates Lys-310 in the RelA/p65 complex in NF-κB, suppressing its pro-inflammatory activity in response to OS, and inhibits the synthesis of pro-inflammatory cytokines or thrombotic factors (ICAM-1, VCAM-1). The protective role of SIRT3 is primarily associated with antioxidant protection through the activation of SOD2 and CAT through deacetylation of the FOXO3a and protection against cardiomyocyte apoptosis. In contrast to SIRT3, SIRT4 decreases mitochondrial MnSOD activity. By interacting with AngII, SIRT4 is also a contributing factor to cardiac hypertrophy. SIRT4 also regulates platelet function and the formation of arterial thrombus, by increasing the expression levels of the fibrinolysis inhibitor PAI-1. ROS, reactive oxygen species; OS, oxidative stress; SIRT1, 3, 4, 5, 6, sirtuin 1, 3, 4, 5, 6; eNOS, nitric oxide synthase 3; ADMA, asymetric dimethylarginine; PRMT1, arginine-1 protein transferase; APE1/Ref-1, purinic/apyrmidinic endonuclease 1/redox factor-1; FOXO3, forkhead box O3; VCAM-1, vascular cell adhesion molecule 1; ICAM-1, intercellular adhesion molecule 1; CAT, catalase; MnSOD, manganese-dependent superoxide dismutase; GPx, glutathione peroxidase; NF-κB, nuclear factor kappa-light-chain-enhancer of activated B cells; Ang II, angiotensin II; PAI-1, plasminogen activator inhibitor-1.

Mitochondrial SIRT5 is highly expressed in the heart, as shown in both animal and human studies, although its function is not fully understood [60]. In the SIRT5 knockout mice, hearts with hypertrophy induced by transverse aortic constriction animal model showed impaired processes of oxidative phosphorylation, TCA, fatty acid oxidation and hypersuccinylation of proteins involved in these processes. It has been shown to decrease NAD^+^/NADH ratio as well as the oxidation of glucose and fatty acids [62,63]. The role of SIRT5 in the regulation of platelet function and the formation of arterial thrombus, which is the cause of acute cardiovascular events, is also indicated. In studies on HAECs treated with SIRT5 si-mRNA, decreased expression levels of the fibrinolysis inhibitor PAI-1 were noted in response to TNFα, through activation of AMPK and reduced phosphorylation of (ERK)1/2 MAP kinase, which indicates the role of SIRT5 in the regulation of fibrinolysis. The SIRT5 knockout mice showed faster thrombus formation in the photochemically injured endothelium without altering platelet function and the clotting cascade. Increased levels of circulating D-dimers and an increased incidence of thrombus embolization have been noted. The above results were confirmed in peripheral blood mononuclear cells of patients with acute coronary syndrome (ACS), which showed significantly higher expression of SIRT5 and PAI-1 compared to the control group without ACS [64]. Figure 3 schematically shows the participation of sirtuins and HIV infection in the development of cardiovascular disorders.

## 6. Insulin Resistance and Diabetes in HIV-Infected Patients and Sirtuins Participation

Type 2 diabetes mellitus (T2DM) is one of the most common concomitant diseases in HIV-infected people, and its occurrence significantly reduces the life expectancy of these patients [65,66]. Data analysis from National Health and Nutrition Examination Survey (NHANES) and Medical Monitoring Project (MMP) from 2009–2010 show a significantly higher risk of developing diabetes in HIV-infected people (*n* = 8610), which was 10.3% vs 8.3%, compared to the general population (*n* = 5604). Among the surveyed people from the HIV group, 52.3% had T2DM, while 3.9% had type 1 diabetes. In addition, compared to the general population, HIV-infected people suffer from diabetes at a younger age and in the absence of obesity. Moreover, the PIs and nucleoside reverse transcriptase inhibitors (NRTIs): zidovudine (AZT), stavudine (D4T), didanosine (DDI) have been indicated as antiretroviral drugs associated with an increased risk of carbohydrate metabolism disorders [67]. In addition, metabolic syndrome (MS), which is a set of risk factors contributing to the development of T2DM and cardiovascular diseases (CVD), including disturbances such as: abdominal obesity, hypertension, insulin resistance, elevated fasting plasma glucose, low high-density lipoprotein (HDL) cholesterol level and high serum triglycerides (TG) is diagnosed more often in HIV-infected people compared to the general population. Lipodystrophy syndrome, characterized by impaired fat metabolism, is also common in HIV-infected patients. This disturbance significantly increases the use of drugs, such as NRTIs and PIs, especially the older generation drugs [68]. PIs inhibit the reversible, uncompetitive glucose transporter type 4 (GLUT-4) in peripheral tissues, resulting in the disruption of insulin secretion and contributing to the development of insulin resistance. PIs also have an indirect effect on GLUT-4 through the up-regulation of heme oxygenase 1 (HO-1), and, as a result, the secretion of pro-inflammatory cytokines, such as IL-8, TNFα, CCL5 and MCP-1, inhibiting GLUT-4 functions and insulin receptor substrate 1 (IRS-1) by activation of c-Jun N-terminal kinase (JNK) and The IκB kinase (IKKβ). Another cause of the development of insulin resistance and T2DM are disorders of the adipocyte differentiation function as a result of PI use, which reduce the activity of phosphatidylinositol 3 kinase (PI3-K) and the intensify β cell apoptosis [69]. In a study of male patients treated with HAART in a regimen containing PIs indinavir (65.2%), nelfinavir (22%), ritonavir (7.6%) or ritonavir+saquinavir (4.6%), Vigouroux et al. [70] reveled that serum adiponectin levels were significantly negatively correlated with body mass index (BMI), waist-hip ratio (WHR), oral glucose tolerance test glycemia and insulinemia, triglycerides, apoB/A1 and hs-CRP levels, and positively correlated with quantitative insulin sensitivity check index (QUICKI) value. Patients treated with the NRTI stavudine had significantly lower serum adipokine levels [70]. This indicates an important role of the applied treatment in the development of these disorders.

Literature data showed that sirtuins affect glucose metabolism in the liver, pancreas, skeletal muscles or adipose tissue through various mechanisms, as described in detail in a number of studies [30,71,72]. The effect of sirtuins activity includes: increased gluconeogenesis, reduced glycolysis, increased lipolysis or increased insulin secretion from β cells, as will be detailed below [31,73,74]. Most of the literature data cover this area and most information is available on SIRT1, SIRT2, SIRT3 and SIRT6.

A main player from the sirtuin family in the modulation of glucose homeostasis is SIRT1, which affects insulin secretion in the pancreatic β cells. One of the target factors influenced by SIRT1 are transcription factors from the FOXO family, which affect the expression of genes related to carbohydrate and lipid metabolism, which will be discussed in detail in the following paragraphs and chapters. SIRT1, deacetylating FOXO1, allows the activation of transcription factors MafA and NeuroD, promoting insulin gene expression in pancreatic β cells [31]. SIRT1 is also the major factor regulating CREB-regulated transcription coactivator 2 (CRTC2) and FOXO1 activity influencing the gluconeogenesis process, depending on the energy supply. It was shown that the overexpression of SIRT1 in mice hepatocytes significantly reduced the amount of CRTC2 following glucagon exposure after 8 h fasting. Exposure of tested mice hepatocytes to SIRT1 inhibitors (sirtinol and nicotinamide) significantly increased CRTC2 activity, intensifying the process of gluconeogenesis. Mice with a hepatic-specific knockdown of SIRT1 gene showed significantly higher levels of CRTC2 and gluconeogenic gene expression compared to wild-type mice during prolonged exposure to glucagon. Deacetylation of CRTC2 by SIRT1 during an extended fasting period (over 18 h fasting) promotes ubiquitin-dependent CRTC2 degradation via COP1 (E3 ubiquitin-protein ligase). The authors concluded that SIRT1 is therefore a factor that regulates the energy balance by influencing the glucose metabolism [75]. Deacetylation of FOXO1 and its co-activator peroxisome proliferator activates receptor gamma coactivator 1 alpha (PGC-1α) by SIRT1, and also increases the transcription of gluconeogenesis genes during longer fasting periods. Deacetylation of PGC-1α by SIRT1 increases its transcriptional activity, increasing the expression of phosphoenolpyruvate carboxykinase (Pck) and glucose-6-phosphatase (G6pc) genes [31].

Another mechanism regulating glucose metabolism by SIRT1 is its effect on protein tyrosine phosphatase (PTP-1B) [69]. PTP-1B is a key phosphatase for the insulin receptor, reverses the action of tyrosine kinases and down-regulates insulin signal transduction. It catalyzes dephosphorylation of the insulin receptor, which in turn protects glucose uptake and blocks further steps in the insulin transduction pathway. SIRT1 modifies the activity of PTP-1B at the chromatin level by deacetylating the Lys9 of histone H3, and reduces its binding in the promoter region for PTP-1B, as shown in an in vitro study of C2C12 murine myotubes by Sun et al. [75] after recombinant HSV encoding SIRT1 infection and induction of insulin resistance by palmitate. The authors noted an increase in SIRT1 expression, insulin receptor (InsR) phosphorylation and glucose uptake after insulin stimulation, and this effect was inhibited by PTP-1B. A decrease in PTP-1B activity in in vivo studies with an increase in SIRT1 expression in the liver of the tested mice during fasting periods was also observed [75].

As mentioned earlier, HAART may negatively affect glucose metabolism, which may contribute to the development of T2DM and insulin resistance. One potential mechanism is the effect on mitochondrial uncoupling protein 2 (UCP2) by PIs. UCP2 is the protein responsible for controlled leakage of protons into the mitochondrial matrix; it lowers the electrochemical gradient of protons, thereby reducing ATP synthesis and glucose stimulated insulin release from β cells. SIRT1, by binding to the promoter for UCP2, inhibits this process, restoring insulin release from pancreatic β cells. The protease inhibitors used in HAART also interact with UCP2. It has been shown that rat insulinoma cells exposed to NFV (5–10 µM) significantly reduce ATP levels, while UCP2 levels are increased [76]. Studies on male Wistar rats orally treated with SQV (333 mg/kg/day) or ATV (133 mg/kg/day) showed significantly reduced levels of pancreatic ATP compared to the control group. Pancreatic UCP2 expression was instead increased in the ATV- and SQV-treated group compared to the control [77]. The above data may indicate the existence of a potential mechanism involving SIRT1, which can be considered to prevent drug-induced negative changes in HIV-infected patients.

Sirtuin 1 also deacetylates the HIF-1 by reducing its transcriptional activity, thereby affecting the glycolysis process by modifying transcription of lactate dehydrogenase (LDH), glucose-6-phosphate isomerase (PGI), phosphofructokinase-1 (PFK-1) or phosphoglycerate kinase 1 (PGK-1). Under hypoxia condition, as a result of lower NAD^+^ concentration, HIF-1 acetylation does not occur, which activates glycolysis. SIRT1 also affects glycolysis processes independent of HIF-1 through deacetylation of phosphoglyceromutase-1 (PGAM-1), which catalyzes the conversion of 3-phosphoglycerate (3-PG) into 2-phosphoglycerate (2-PG) in glycolysis process, and thus its deactivation. In in vitro studies, Hallows et al. [78] showed increased levels of PGAM-1 acetylation in HEK293 cells under the influence of an SIRT1 inhibitor (10 mM sirtinol) [78].

Sirtuin 2 is also indicated as an important modulator of insulin resistance development. SIRT2 stabilizes and deacetylates PEPCK1 by inhibiting the action of E3 ubiquitin-protein ligase component N-recognin 5 (UBR5). PEPCK1 converts oxaloacetate into phosphoenolpyruvate in the gluconeogenesis process. Deacetylation of PEPCK1 prevents degradation by ubiquitin-proteasome pathway, promoted by acetylation of Lys70, Lys71 and Lys594 by p300 acetyltransferase. In an animal study, it was found that SIRT2 knockdown decreased PEPCK1 level by 70% without changing the amount of its mRNA, and there was a 35% decrease in glucose levels in mice with SIRT2 knockdown [72]. SIRT2 has been shown to be down-regulated in mice with insulin resistant hepatocytes and accompanied by increased ROS accumulation and mitochondrial dysfunction. SIRT2 increases the expression of fusion-related protein mitofusin 2 (Mfn2) and downregulates mitochondrial-associated dynamin-1-like protein (Drp1), which improves mitochondrial function by enhancing the mitochondrial fragmentation process, and also enhances the expression of mitochondrial transcription factor A (TFAM), resulting in increased mitochondrial mass. Moreover, SIRT2 expression was negatively correlated with OS, obesity and insulin resistance in human PBMCs [79]. SIRT2 also regulates redox homeostasis by deacetylating FOXO3a, which increases MnSOD expression. Deacetylation of Lys403 of G6PD by SIRT2 also increases the production of NADPH and the reduced form of glutathione (GSH), which points to the antioxidant action of SIRT2 [71]. In adipocytes, down-regulation of SIRT2 reduces the degree of PGC-1α deacetylation and fatty acid catabolism. In visceral adipose biopsies of obese humans (BMI> 30 kg/m^2^), increased expression of HIF1-α and decreased expression of SIRT2 were observed. An inverse relationship was observed in lean individuals (BMI <25 kg/m^2^) [80]. SIRT2 is also considered to inhibit adipogenesis through deacetylation of FOXO1; as a result, it promotes its binding to peroxisome proliferator-activated receptor α (PPARα), reducing its transcriptional activity. After 24 h of fasting, mice showed increased expression of SIRT2 in white adipose tissue. Mice exposed to 5 °C (temperature as an inducer) for 12 h showed increased expression of SIRT2 in brown adipose tissue. Increased expression of SIRT2 was also noted in 3T3-L1 cells treated with β-adrenergic agonist, isoproterenol [81]. The above data indicate the key role of SIRT2 in the development of obesity, which is one of the main causes of the development of T2DM and other metabolic disorders connected with the course of HIV infection.

Sirtuin 3 is a factor that affects energy homeostasis primarily in skeletal muscles. It is the major mitochondrial deacetylase. The source of energy for skeletal muscles can be both glucose and lipids. They are characterized by metabolic flexibility and have the ability to change the energy substrate and obtain energy from glucose or lipid oxidation. A reduced amount of CREB, PGC-1α and CS and AMPK was found in SIRT3 deleted mice [82,83]. Jing et al. [84] showed that SIRT3 also deacetylates ATP synthase, affecting glucose homeostasis in skeletal muscle. This indicates an important role of this sirtuin in the development of metabolic disorders in the course of HIV. The pyruvate dehydrogenase (PDH) enzyme, which controls glucose use as a main energy source in skeletal muscles, is also affected by SIRT3 action. PDH is considered as a decisive factor in a complex mechanism of carbohydrate saving in the period of low glucose supply, and during the switch to use fats as the main source of energy. It catalyzes the oxidative decarboxylation of pyruvate in acetyl-CoA, allowing the incorporation of the glycolysis-pyruvate product in TCA. It has been shown that mitochondrial SIRT3 regulates the process of post-translational acetylation of the pyruvate dehydrogenase E1α subunit and decreases enzyme activity, which leads to a reduction in glucose oxidation and the use of fats as the main source of energy [85]. In SIRT3 knockout mice muscle mitochondrial lysates, PDH activity was significantly lower compared to WT mice. The increased amount of lactate and pyruvate in the muscles from fed SIRT3 KO mice, along with decreased PDH activity and a decreased amount of acylcarnitines and amino acids compared to WT suggests that SIRT3 deficiency causes changes in the metabolic profile of muscles towards the use of fatty acids and amino acids, which emphasizes the important role of SIRT3 in maintaining muscle energy homeostasis [83,86]. SIRT3 also deacetylates Lys 42 of a key enzyme responsible for liver β-oxidation of fatty acids-long-chain specific acyl-CoA dehydrogenase (LCAD), increasing its activity. Mice with SIRT3 knockdown starved for 48 h showed a reduction in fatty acid oxidation in brown adipose tissue and liver compared to the control group (WT mice). It was also shown that the SIRT3 knockdown mice showed an intolerance to cold exposure, which is associated with impaired oxidation of fatty acids and metabolic stress [35,87]. Agrawal et al. [36] demonstrated that WT mice injected with Vpr viral protein showed reduced β-oxidation of fatty acids in the liver by about 45%, associated with decreased expression of enzymes regulated by PPARα among others, with lower expression of LCAD mRNA, the activity of which also regulates SIRT3. This indicates a possible interaction of this sirtuin with viral Vpr protein [36]. The effect of PPARα and SIRT3 on fat metabolism and the mechanistic action of Vpr on metabolism will be described in detail later in this article.

**Figure 4:**
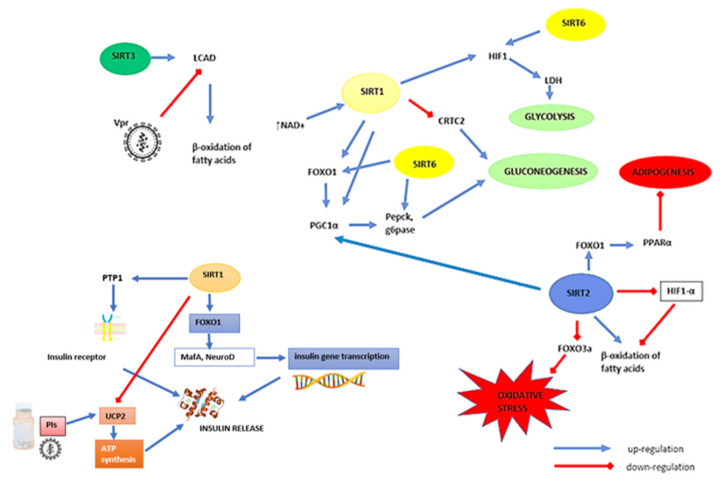
Scheme of connection between sirtuins, carbohydrate metabolism and HIV infection. SIRT1, by deacetylation of FOXO1, activates the transcription factors MafA and NeuroD and increases the expression of the insulin gene in pancreatic β cells. By deacetylation of PT-1B, SIRT1 reduces its insulin signal transduction and insulin release inhibitory activity. SIRT1 binds to the promoter for UCP2, inhibits the process of reducing ATP synthesis and restores insulin release from pancreatic β cells. Protease inhibitors act the opposite of SIRT1, they increase the activity of UCP2. SIRT1 down-regulates CRTC2, inhibiting gluconeogenesis during an extended fasting period. Deacetylation of FOXO1 and its co-activator PGC-1α by SIRT1, increases the transcription of gluconeogenesis genes. SIRT1 and SIRT6 deacetylates the HIF-1 by reducing its transcriptional activity, thereby intensifying glycolysis. SIRT2 regulates redox homeostasis by deacetylation of FOXO3a, which increases MnSOD expression and the reduced form of glutathione. In adipocytes, SIRT2 deacetylates PGC-1α, intensifying fatty acid catabolism and gluconeogenesis. SIRT2 also inhibits adipogenesis through deacetylation of FOXO1, promoting its binding to PPARα and reducing its transcriptional activity. SIRT3 deacetylates a key enzyme responsible for liver β-oxidation of fatty acids, LCAD, increasing its activity. Vpr viral protein reduce β-oxidation of fatty acids in the liver and decreases expression of LCAD. The effect of SIRT6 on glucose metabolism is associated with the inhibition of gluconeogenesis via the PGC-1α, which in turn inhibits the expression of PEPCK and glucose 6-phosphatase, associated with gluconeogenesis. SIRT6 also acts by deacylating FOXO1-inhibiting gluconeogenesis. SIRT1,2,3,6, sirtuin 1,2,3,6; HIF1, hypoxia inducible factor-1; LDH, lactate dehydrogenase; CRTC2, CREB regulated transcription coactivator 2; FOXO1, forkhead box protein O1; FOXO3a, forkhead box protein O3a; IL-6, interleukin 6; LCAD, long-chain specific acyl-CoA dehydrogenase; PC1α, peroxisome proliferator activated receptor gamma coactivator 1 alpha; STAT3, signal transducer and activator of transcription 3; UCP2, mtochondrial uncoupling protein 2; PTP1, protein tyrosine phosphatase 1; PPAR-α, peroxisome proliferator-activated receptor alpha; HIF1-α, hypoxia-inducible factor 1-alpha.

The literature data also indicate the role of SIRT6 in glucose metabolism disturbances, which may also be associated with the development of diabetes, which, as we know, is associated with HIV course. Xiong et al. [86] observed a reduction in glucose-stimulated insulin secretion (GSIS) in mice with knockdown of the SIRT6 gene by nearly 50% compared to the control group. Changes in mitochondria function and lower ATP levels were also observed, which resulted in disturbances in the transport of electrons in the respiratory chain; disturbances in plasma membrane depolarization and post-depolarization as well as calcium flux in β-cells were also observed. The effect of SIRT6 on glucose metabolism is associated primarily with the inhibition of gluconeogenesis. This process takes place via the PGC-1α, indirectly, by the activation of general control non-repressed protein 5 (GCN5), which subsequently acetylates PGC-1α, and in turn inhibits the expression of phosphoenolpyruvate carboxykinase C (PEPCK-C) genes and glucose 6-phosphatase, associated with gluconeogenesis. SIRT6 also acts by deacylating FOXO1-inhibiting gluconeogenesis, as described above [31,87]. Sirtuin 6 has been shown to influence the IRS/PI3K/AKT signaling pathway. As a result, inhibition of IRS phosphorylation leads to down-regulation of AKT (which also reduces expression of FOXO1, GSK3, mTORC1), insulin signaling and decreased glucose uptake. SIRT6 also inhibits GLUT-1 and -4 expression, which prevents hypoglycemia [88]. Old generation protease inhibitors (mainly ritonavir and indinavir) also interact with GLUT-4, impairing glucose transport, as mentioned above, which is one of the causes of disorders of carbohydrate metabolism in patients with HIV [89]. Bresciani et al. [89] have shown in the 3T3-L1 adipocyte cell model that exposure to lopinavir/ritonavir caused overexpression of miRNA-218 and the associated lower expression of lipin-1, the main factor determining GLUT-4 expression [89]. SIRT6 regulates glycolytic enzymes (PDK1, PFK1 and LDH) and HIF-1α activity in skeletal muscle, which also leads to the suppression of GLUT1 and GLUT3 [89,90]. Glycolysis is another mechanism regulated by SIRT6. Studies have shown that SIRT6, similarly to SIRT1, deacetylates and thereby inhibits HIF-1 and H3K9, which increases the expression of the genes responsible for glycolysis [86,90]. Figure 4 shows schematically the role of sirtuins (SIRT1, SIRT2, SIRT3 and SIRT6) in the development of carbohydrate disorders, taking into account the impact of HIV infection and antiretroviral therapy.

## 7. Bone Metabolism Disturbances in HIV-Infected Patients and the Role of Sirtuins

Negative changes associated with bone metabolism are common in HIV-infected patients. An open prospective cohort study (*n* = 5826) conducted between 2000–2006 as part of the HIV outpatient study (HOPS) showed that fracture rates and relative proportion of fragility fractures were significantly higher in HOPS patients compared to the general population [91]. Gibellini et al. [92] showed that in plasma of HIV-infected men, levels of markers associated with bone formation, such as osteoprotegerin (OPG), receptor activator of NF-kappa b-ligand (RANKL), and the TNF-related apoptosis-inducing ligand (TRAIL) were significantly higher in the HIV-infected group compared to the control group [93]. The problem of reduced bone density is primarily caused by the negative impact of the Vpr on RANKL. Up-regulation of RANKL causes increased osteoclast formation and lower levels of OPG (RANKL inhibitor), the main factors determining bone metabolism and the degree of bone resorption [91,94]. In a cross-sectional study, statistically significant lower OPG production by T cells and higher T cell RANKL production was shown in HIV-infected patients, compared to healthy individuals [94]. Titanji et al. [95] showed significantly lower OPG expression and higher RANKL expression in B cells of HIV-positive patients, compared to negative controls. The authors also found a correlation between RANKL/OPG ratio in B cells and T-score (difference between the current bone density value and the theoretical mean peak bone mass), Z-score (difference between the current bone density value and the age-appropriate theoretical mean normal value) and total bone mineral density (BMD) in areas with the highest fracture risk: hip and femoral neck [95].

A higher risk of osteoporosis is associated not only with the HIV infection itself, but HAART also has an impact. In a cohort study (*n* = 40.115), Womack et al. [93] found significant positive correlations between increased fragility fracture risk and current PI use among HIV-infected patients [93]. The decline in BMD is independent of the type of HAART, however, PIs were considered to be the most osteotoxic agents. Some studies show that PIs in particular contribute to bone metabolism disorders. In patients treated with PIs, bone density in the lumbar spine after one year decreased significantly compared to other therapeutic regimens [96,97]. In vitro studies on human osteoblast cultures have shown that PIs change the expression of some genes responsible for calcium deposition in bones, they reduce alkaline phosphatase (ALP) and runt-related transcription factor- 2 (Runx- 2). Down-regulation in the expression of tissue inhibitors of metalloproteinases-3 (TIMP-3), responsible for osteoblast differentiation and matrix development processes, has been noticed in NFV and IDV [98]. Cozzolino et al. [99] have shown that among the analyzed HIV cases, a decrease in BMD by more than 5% was observed in 31% of patients after 4 years of virologic suppression, as a result of combined antiretroviral therapy (cART). In addition, in the National Health and Nutrition Examination Survey (NHANES), a significantly higher incidence of lower BMD in the femoral neck was demonstrated compared to controls (47% vs. 29%). PIs probably have a negative effect on vitamin D3 metabolism, as they can inhibit 25-α-hydroxylase 1a, which converts 25-hydroxyvitamin D into its active form, 1,25-dihydroxyvitamin D [99]. Another mechanism may be increased bone resorption caused by proximal renal tubular damage by tenofovir (TDF), leading to hypophosphatemia and increased parathyroid hormone release. In addition, tenofovir phosphate compounds interact directly with osteoblasts and osteoclasts, contributing to increased bone resorption [98,100]. Negredo et al. [100] showed significantly reduced BMD in HIV-infected patients, which was connected with changes in TDF to abacavir (ABC) after a 48-week period, and was manifested by a statistically significant lower level of some serum bone markers: collagen type 1 cross-linked C-telopeptide (CTX), type I procollagen N-terminal propeptide (P1NP), osteocalcin and higher sclerostin levels [100].

The influence of sirtuins on bone metabolism is increasingly indicated in HIV-infected patients. Choi et al. [101] have shown that SIRT1 is a deacetylating agent of SRY-Box transcription factor (SOX2), the main factor maintaining the self-renewal and ability to differentiate mesenchymal stem cells (MSCs), that is also observed in osteoblasts. SOX2 maintains stem cells by regulating the expression of Dickkopf-related protein 1 (DKK1). The SIRT1/SOX-2 axis has been shown to regulate the differentiation or regeneration of MSCs. Deacetylation of SOX2 by SIRT1 inhibits its transport from the nucleus to cytosol, degradation by proteasomes and ubiquitination, as demonstrated in in vitro studies on bone marrow mesenchymal stem cells (BM-MSCs) [101]. A positive effect of resveratrol (RSV), an SIRT1 activator, on maintaining SOX2 activity has also been demonstrated in cultured MSCs, which retained their ability to regenerate and proliferate under exposure to RSV (1 µM), compared to unexposed cells [102]. The positive effect of RSV was also shown in an animal model, where it was administered in three doses (5, 25 and 45 mg/kg/day) in female rats after ovariectomy. Statistically significant higher BMD was shown, compared to the post-ovariectomy group that did not receive RSV at medium and high doses [103]. The same authors, in an in vitro study on BMSCs, confirmed that RSV significantly increased the level of SIRT1 expression, while reducing NF-κB p65 and p-IκBα compared to controls (untreated with resveratrol). As confirmed by siRNA-SIRT1 transfection, the beneficial effect of resveratrol is due to a modification of the SIRT1/NF-κB signaling pathway activity. The authors also showed that SIRT1 activation contributed to the induction of BMSC differentiation towards osteoblasts [104]. Similar results were found by Zainabadi et al. [103] in mice treated with SIRT1 synthetic agonist, SRT1720 (100 mg/kg/day). There was an almost 30% increase in femoral bone mass in SRT1720-treated mice, compared to only vehicle treated mice [103]. Expression of SIRT1 was confirmed in BM-MSC and primary bone marrow cultures. Cohen-Kfir et al. [104] in an animal model (SIRT1+/− and wild type (WT) mice) as well as a BM-MSC cell model showed significantly reduced bone formation in SIRT1+/− mice compared to WT. The authors revealed a statistically significantly lower mRNA expression of sialoprotein, osteocalcin and type 1 collagen in SIRT1 +/− BM-MSC. The study additionally showed that Sirt +/− mice significantly increased the expression of the sclerostin (SOST) gene, one of the major osteoblastogenesis inhibitors [104]. Similar results were obtained in vitro when examining human osteoarthritis subchondral osteoblasts, which also showed increased expression of SOST and decreased SIRT1 expression compared to non-osteoarthritic subchondral osteoblasts [105]. In studies conducted by Mora et al. [106], statistically significant lower serum concentration of Wnt antagonists sclerostin and DKK-1 were found in HIV-infected patients compared to healthy people [106]. The impact of HAART on sclerostin levels was also confirmed by studies of Erlandson et al., who showed that the median value of sclerostin was higher before HAART implementation compared to post-HAART in HIV-infected patients [107].

**Figure 5:**
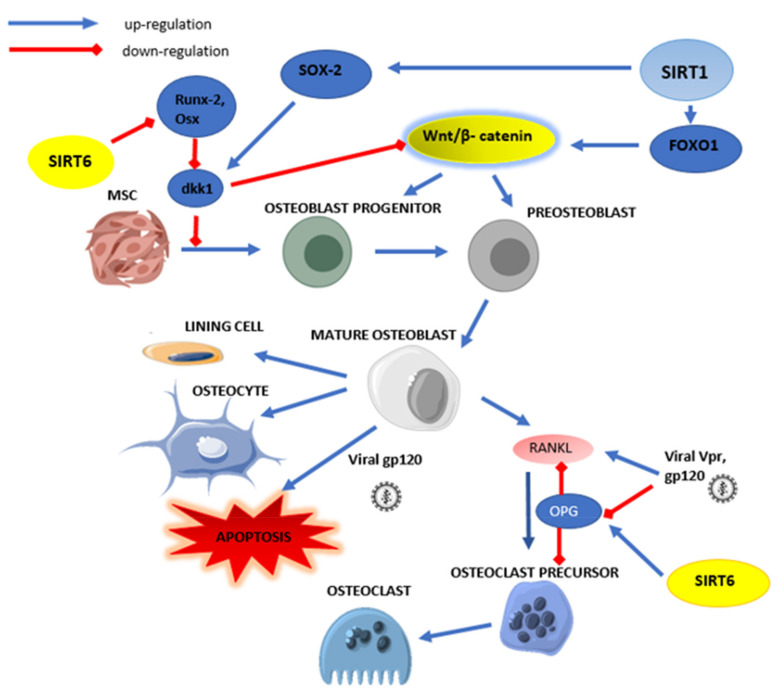
Scheme of connection between sirtuins, HIV infection and bone metabolism. SIRT1 deacetylates and up-regulates SOX2, the main factors maintaining the self-renewal and ability to differentiate mesenchymal stem cells in osteoblasts. FOXO1 deacetylation by SIRT1, up-regulates the differentiation of preosteoblast and osteoblast progenitor into osteoblasts. SIRT6 down-regulates Runx2 and Osx genes, responsible for inhibiting blastogenesis and the transition of osteoblasts to osteocytes. SIRT6 up-regulates OPG-RANKL inhibitor, decreasing bone resorption. RANKL, receptor activator for nuclear factor κ B ligand; OPG, osteoprotegerin; FOXO1, forkhead box protein O1; SOX-2, SRY-Box transcription factor 2; Runx2, runt-related transcription factor 2; DKK-1, Dickkopf-related protein 1; MSC, mesenchymal stem cells.

The other enzyme from the sirtuin family that is important in bone remodeling disturbances is SIRT6. Zhang et al. [108] showed abnormal bone remodeling formation and resorption processes in mice with SIRT6 knockdown. Genes in osteoblasts are indicated as potential mechanism changes in the expression of runt-related transcription factor 2 (Runx2) and Osterix (Osx). In the absence of SIRT6, there is increased acetylation of histone H3K9 in the promoter region for Runx2 and Osx, responsible for inhibiting blastogenesis and the transition of osteoblasts to osteocytes. The authors suggest that SIRT6 may be considered as an important determinant of osteoblastogenesis. Increased expression of OPG and DKK1 has also been observed in SIRT6 deficiency, which causes disturbances in osteoblast and osteoclast differentiation. Studies also show that SIRT6 is a positive regulator of osteogenic differentiation. SIRT6 regulates osteogenic differentiation via one bone morphogenetic protein (BMP) signaling in a 300/CBP-associated factor (PCAF)-dependent manner, which, when activated, binds to the bone morphogenetic protein 2 (BMP2) and bone morphogenetic protein 4 (BMP4) promoters, which in turn activate Runx2, a key osteogenic differentiation factor [108]. Sirtuin 6 also controls IGF-1-mediated bone resorption, affecting hypoxia-induced osteoblast apoptosis. Under hypoxia, higher pro-inflammatory cytokine production and increased OS and glycolysis can be observed. Studies conducted on human osteoblast cells (HOB) have shown that SIRT6 inhibits the above processes. In a culture under hypoxia cells, increased expression of lactate dehydrogenase (LDH) and lactate as well as increased reactive oxygen species (ROS) generation was observed. Overexpression of SIRT6 reduced the above changes and decreased expression of pro-inflammatory factors IL-6, IL-1β, TNFα, confirming the role of SIRT6 in preventing inflammatory bone resorption [109]. Figure 5 schematically shows the effect of sirtuins (SIRT1 and SIRT6) on bone metabolism during HIV infection.

## 8. Kidney Diseases in HIV-Infected Patients and the Role of Sirtuins

Kidney diseases are diagnosed more often in HIV patients compared to the general population. These diseases include acute kidney injury (AKI), HIV-associated nephropathy (HIVAN), comorbid chronic kidney disease (CKD), HIV immune complex kidney disease (HIVICK), thrombotic microangiopathy and treatment-related kidney toxicity predisposed to CVD, heart failure and end-stage renal disease (ESRD) [110,111]. In the years 1996–2006, CKD became one of the most common causes of death in people infected with HIV and one of the most common comorbidities in HIV-infected patients, affecting 2–10% of infected patients in European and American countries [111]. CKD is also a risk factor for the development of AKI in HIV-positive patients. Other risk factors include glomerular filtration rate (eGFR) < 60 mL/min/1.73m^2^, proteinuria > 30 mg/dL, diabetes, hypertension, HCV-co infection [112]. Proximal renal tubular dysfunction, albuminuria and renal impairment are more prevalent in HIV-positive people compared to uninfected controls [113]. In a cross-sectional study conducted on 2339 HIV-positive patients, CKD was diagnosed in 13.3% of them, 12.6% had albuminuria and 4.6% had eGFR < 60 mL/min/1.73m^2^ [114]. Additional risk factors for kidney disease are age, hypertension, diabetes mellitus, nadir CD4+ cell count <200 cells/µL, use of tenofovir disoproxyl fumarate (TDF) and use of TDF with ritonavir-boosted PIs [111,115]. Mitochondrial dysfunction and depletion of mitochondrial DNA are considered to be potential mechanisms of TDF toxicity, as a result of drug accumulation caused by dysfunction or inhibition of the multidrug resistance-associated protein-4 (MRP4) transporter. It has been shown that incidence of CKD significantly increases with the next year of exposure to TDF (incidence rate ratio [IRR]: 1.14), ritonavir-boosted atazanavir (IRR: 1.20) and ritonavir-boosted lopinavir (IRR: 1.11), but not other ritonavir-boosted PIs or abacavir [116]. The virus utilizes lipid rafts, and through their mediation enters the cell [117]. HIVAN, the most common kidney disease in infected patients, is characterized by podocyte collapse, hyperplasia, tubulointerstitial and glomerular damage, focal glomerular sclerosis and proteinuria. Mitochondrial dysfunction and changes in their morphology have been shown to be the cause of HIVAN, which is associated with the expression of viral proteins Nef, Vpr and Tat, ROS generation and apoptosis of renal cells. Tg26 mice kidneys showed significantly lower expression of PGC-1α-promoting mitochondrial biogenesis; mitofusin-2 (MFN2), a mitochondrial membrane protein responsible for mitochondria fusion; and PARKIN-E3 ubiquitin ligase, which induces the autophagic degradation of mitochondria [118,119].

It was shown that renal tubular cells express HIV proviral genes, even with undetectable viremia in the blood. Podocytes are also a reservoir of the virus. The suggested mechanism for the entry of the virus into renal cells is via its interaction with the DEC-205 (CD205) receptor secreted at the tubular cells. The binding of viral gp120 to DEC-205 has been demonstrated. Expression of C-X-C motif chemokine receptor 4 (CXCR4) was demonstrated in podocytes. In podocytes, endothelial and tubular cells, SIRT1 is indicated as an important agent in kidney disturbances. Deacetylation of PGC-1α by SIRT1 increases its activity as a cofactor for the transcriptional repressor protein YY1, promoting mTOR mediated growth and division of mitochondria. PGC-1α also activates PPARα and ERRs that regulate the processes of e.g., β-oxidation of fatty acids, affecting mitochondrial processes. Lempiäinen et al. [120] found in Wistar rats kept under calorie restriction (energy intake 70%) with ischemia/reperfusion (I/R) kidney injury showed improved renal function by attenuating the nitrative stress caused by I/R, which resulted in preventing tubular necrosis compared to control group (rats with I/R without calorie restriction). The authors indicated that during kidney injury, the expression of PGC-1α, AMPK, SIRT1 and eNOS was reduced. Calorie restriction partially inhibited this effect by increasing SIRT1 expression [120]. Rats treated with SIRT1 activator SRT1720 had significantly increased renal ATP levels, which prevented mitochondrial mass decline, increased PGC-1α expression, decreased apoptosis and prevented renal histologic architecture damage, compared to vehicle treated animals [121]. Mice with AKI induced by cisplatin showed mitochondrial abnormalities in tubular cells with a reduced SIRT3 mRNA expression and protein level. Treatment of the tested mice with AMPK activator, AIKAR (5-aminoimidazole-4-carboxamide-1-β-D-ribofuranoside) and antioxidant ALKAR (agent acetyl-l-carnitine) improved renal function through restoring SIRT3 activity. Furthermore, in vitro studies on renal proximal tubular epithelial cells (RPTECs) damaged by cisplatin showed reduced SIRT3 expression compared to controls’ mitochondrial fission. It has been shown that SIRT3 prevents the recruitment of dynamin-related protein 1 (DRP-1) and the up-regulation of mitochondrial fission factor (MFF) and PTEN-induced kinase 1 (PINK1) in mitochondria, preventing mitophage processes and preserving their integrity. In addition, SIRT3 has a positive effect on the potential of mitochondrial membranes and prevents depolarization because it increases the activity of optic atrophy 1 (OPA1) profusion protein, thus preventing the fragmentation and cleavage of mitochondria as well as the loss of organelles in tubular epithelial cells, which suggests the renoprotective effect of SIRT3 [122].

SIRT1 has been shown to regulate the functioning of the RAS system with a positive effect on blood pressure and kidney function. SIRT1 binds to the promoter for angiotensin-converting enzyme 2 (ACE2) and increases its expression, promoting the conversion of Ang to AngII, preventing vasoconstriction, sodium reasorption and decreased hemodynamics of the kidneys [123]. The activation of the RAS is also considered a factor in the development of HIVAN. Reducing the production of AngII has been shown to significantly slow the progression of HIVAN [124]. In studies on Tg26 mice, the phenotype of HIVAN (sclerosed or collapsed) has been shown to be associated with RAS activation. After 4 weeks of subcutaneous administration of normal saline (control group), ACE inhibitor captopril (5 mg/kg/day), tekturna-renin inhibitor (50 mg/kg/day) and angiotensin receptor (AT1) antagonist telmisartan (0.3 mg/kg/day) to tested animals, significantly lower amounts of sclerosted glomeruli and significantly lower levels of blood urea nitrogen were observed in all study groups, indicating improved renal function compared to the control group. The amount of collapsed glomeruli remained unchanged [124]. The above data may indicate the renoprotective effect of SIRT1, also in the course of HIVAN. Other studies have shown a protective effect of SIRT1 on podocytes, which is crucial for the proper functioning of the glomeruli by preventing excessive OS and apoptosis, as well as the subsequent damage due to deacetylation and a decrease in the activity of the previously described transcription factors: NF-κB, STAT3, FOXO4, p53 and PGC-1α. Deacetylation of FOXO4 by SIRT1 prevents activation of BCL-2 gene transcription and the induction of apoptosis in podocytes and tubular cells. Increased accumulation of acetylated FOXO4 has been demonstrated as a result of decreased SIRT1 activity in podocytes in the course of hyperglycemia in diabetic kidney disease. Weakened expression of SIRT1 in cultured podocytes isolated from diabetic mice was caused by an increased amount of advanced glycation end products (AGEs) characteristic of long-term hyperglycemia [125]. Increased OS and DNA damage have been demonstrated in HIV-infected podocytes as a result of the activation of the p66ShcA/FOXO3a pathaway. Activation of p66ShcA creates a complex with FOXO3a and causes its sequestration to the cytosol, which inhibits the expression of antioxidant genes of MnSOD or genes regulating the cell cycle, including cyclin-dependent kinase inhibitor (CDKI) [126].

It has been shown that the up-regulation of p66Shc protein is correlated with renal damage both in vivo and in vitro [127]. Presumably, SIRT1 deacetylates p66ShcA in Lys 81, preventing its pro-oxidative effects, as demonstrated in aortic endothelial cells of diabetic mice [128]. The existence of a similar mechanism in the kidneys has been indicated by studies conducted by Yang et al. [127], who analyzed renal tissue samples of renal biopsies from diabetic nephropathy patients and controls (non-diabetic renal diseases patients). The expression of p66Shc increased by about 50% and SIRT1 expression decreased by approximately 30% in glomeruli and renal tubules of patients compared to controls. The authors also showed decreased p-AMPK and SIRT1 expression compared to controls and increased expression of p66Shc in the kidneys of diabetic mice. After administration of probucol—an antioxidant drug—the above changes were reversed. In vitro studies using human proximal tubular cells (HRCE) exposed to high glucose conditions (30 mM, for 1–6 h) showed that probucol activates AMPK/Sirt1 pathway and inhibits p66Shc expression and ROS generation by phosphorylation of AMPK in diabetic nephropathy [127]. SIRT1 is also an activating factor of FOXO3, which confirms its antioxidant effect. Attenuated renal function and glomerulosclerosis has been demonstrated in rats with diabetic nephropathy via activation of SIRT1 through RSV treatment [129].

**Figure 6:**
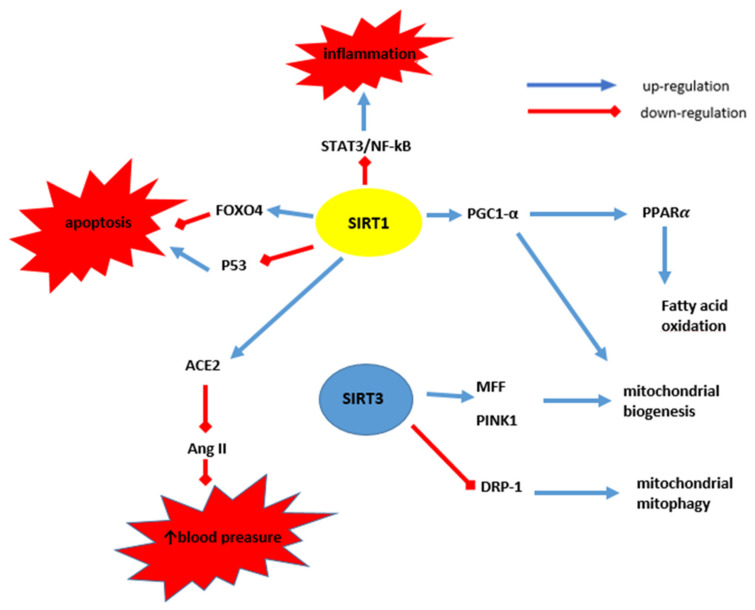
Scheme of connection between sirtuins, kidney disturbances and HIV infection. SIRT1 decreases the activity of NF-κB, STAT3, FOXO4, p53 and PGC-1α in podocytes, preventing excessive OS, inflammation and apoptosis. Deacetylation of PGC-1α by SIRT1 increases its activity, promoting growth and division of mitochondria. PGC-1α also activates PPARα and regulates the processes of β-oxidation of fatty acids, affecting mitochondrial processes. SIRT1 binds to the promoter for angiotensin-converting enzyme 2 (ACE2) and increases its expression, promoting the conversion of Ang to AngII, and regulates the functioning of the RAS system with a positive effect on blood pressure and kidney function. Deacetylation of FOXO4 by SIRT1 prevents activation of BCL-2 gene transcription and the induction of apoptosis in podocytes and tubular cells. SIRT3 prevents mitophage processes and preserves mitochondria integrity by the down-regulation of DRP-1 and the up-regulation of MFF and PINK1. SIRT 1, 3, sirtuin 1, 3; FOXO4, forkhead box O4; NF-κB, nuclear factor kappa-light-chain-enhancer of activated B cells; Ang II, angiotensin II; ACE2, angiotensin-converting enzyme 2; PPARα, peroxisome proliferator-activated receptor alpha; PGC1-α, peroxisome proliferator-activated receptor gamma coactivator 1-alpha; STAT3, signal transducer and activator of transcription 3; MFF, mitochondrial fission factor; PINK1, PTEN-induced kinase 1; DRP-1, dynamin-related protein; OS, oxidative stress.

Another pathomechanism in podocytes is the reorganization of the cytoskeleton through the influence of SIRT1 on cortactin. Deacetylated cortactin is transported from the nucleus to the cytosol and is responsible for maintaining the cytoskeletom integrity and the proper functioning of the split membrane. It was noted that in SIRT1 (pod^−/−^ mice (mice with podocyte-specific SIRT1 knockout), podocyte damage (induced by protamine sulfate) was more severe compared to podocyte injury in WT mice. Analysis of isolated glomerules showed significantly reduced expression of podocyte-specific proteins, WT-1 (Wilms’ tumor 1 protein), nephrin, synaptopodin, in SIRT1^−/−^ mice compared to WT. Moreover, SIRT1^−/−^ mice podocytes showed increased F-aktin accumulation and albuminuria in comparison with WT mice with glomerular disease, which indicates an increased damage to the cytoskeleton in the absence of SIRT1 [130]. Increased NF-Kb p65 and STAT3 acetylation in HIVAN has been demonstrated in parallel with decreased SIRT1 expression in the glomeruli of mouse and human HIVAN kidneys. In vivo studies on Tg-26 mice, following administration of BF175, a SIRT1 agonist, decreased albuminuria and expression of inflammatory markers. In the course of HIV infection, miR-34 leads to a decrease in SIRT1 expression in the kidney, which contributes to the development of inflammation following kidney injury [131]. Figure 6 shows schematically the participation of sirtuins in the development of kidney diseases in HIV-infected patients.

## 9. NeuroAIDS in Aging HIV Population and Sirtuin Participation

As shown by the data, the number of people infected with HIV over the last 50 years is over 10%, and in more developed regions it reaches up to 50%, and it is estimated that it will grow [132]. Accelerated aging in HIV-positive patients is associated with the induction of oxidative stress; mitochondrial dysfunction; cell cycle arrest and induction of the state of chronic immune activation in the course of infection; viral proteins, such as Nef or Tat; or chronic antiretroviral therapy [133]. One of the characteristics of accelerated cellular aging is shortening telomere length. Decreased absolute length of peripheral blood leukocytes’ telomere length has been demonstrated in HIV-positive patients compared to uninfected controls. Telomere length was inversely correlated with the CD4 nadir + cell count and duration of infection [134]. It has also been shown that sirtuins affect telomere length; prevent their abrasion and promote DNA damage repair; preserve genome integrity; and stabilize the chromatin structure [135]. In liver specific telomerase knockout mice, telomere shortening results in repression of all sirtuins in a p53-dependent manner: SIRT1, SIRT2, SIRT6 by miRNA-34a, 26a and 145a, and SIRT3, SIRT4, SIRT5 by peroxisome proliferator-activated receptor gamma coactivator 1-alpha and beta (PGC-1α/β). On the other hand, NAD + supplementation caused telomere stabilization through overexpression of sirtuins [136]. SIRT6 is considered a critical regulator of DNA repair in telomeric regions, and is a positive regulator of longevity through its influence on the metabolic and telomere functions [137]. Sirtuins, by regulating many signaling pathways, such as FOXOs, AMP-activated protein kinase, insulin/IGF-1 signaling and others, are considered to be key lifespan regulators delaying cellular aging [8,31]. In the aging HIV population, as stated in the preceding paragraphs, there is a greater risk of age-related morbidities, such as cardiovascular diseases or metabolic disorders, including neurodegenerative diseases [1].

NeuroAIDS, which is a collection of cognitive, motor and autonomic disorders associated with HIV infection, is still a serious problem in medical practice among HIV-positive patients. The central nervous system (CNS) is the viral reservoir that is occupied in the initial stage of infection, leading to HIV-associated neurocognitive disorders (HAND) [138]. Current treatment regimens have good CNS penetration, and rapid implementation of cART effectively prevents more severe forms of HAND [139]. As a result of the introduction of cART, the incidence of diseases such as AIDS dementia (AD), myelopathy or sensory neuropathy has significantly decreased [140]. However, about 50% of HIV-infected patients suffer from one of the milder forms of HAND, the most common being asymptomatic neurocognitive impairment (ANI) and mild neurocognitive disorder (MND) [132]. HIV RNA is still detected in the cerebrospinal fluid (CSF) of virologically suppressed patients [141].

Neurotoxicity as a result of HIV is mainly related to the infiltration of infected macrophages, lymphocytes and multinucleated giant cells. As a result of infiltration of virus-activated cells of the immune system into endothelial cerebral vessels, the integrity of the blood-brain barrier (BBB) is damaged, facilitating further migration of infected cells. The damage is perpetuated by the release of pro-inflammatory cytokines and toxins causing neuroinflammation of macrophages. Significantly increased concentrations of neopterin—a macrophage activation marker—have been shown in patients virologically suppressed by cART [142]. Moreover, inflammatory changes in CSF are present in HIV-positive patients, even in asymptomatic stages of infection [139]. Despite effective treatment and undetectable viremia, viral proteins (Tat, Gp120 and Vpr) are active in the CNS and interact with CXCR4 and CCL5 coreceptors, causing activation of microglia, astrocytes and macrophages. The viral proteins Tat, gp120 and Vpr induce apoptosis of neurons through increased expression of TNF-α, IL-6 and IL-1 and ROS production. The presence of Tat protein has been demonstrated in CSF in HIV-infected people successfully treated with antiretroviral therapy [143].

All seven sirtuins are widely distributed in the CNS structures. SIRT1 and SIRT2 are most commonly expressed, mitochondrial sirtuins (SIRT 3, SIRT4, SIRT5) are expressed to a lesser extent and SIRT6 at the lowest degree. SIRT1 expressions are observed mostly in the cerebellum, hippocampus and hypothalamus neurons, SIRT2 in the spinal cord and brainstem, cerebellum cortex, striatum and hippocampus [144].

SIRT1 is considered to be one of the essential factors in stabilizing the genome in neurons. In vitro studies on cultured mice SIRT1 knockout neurons showed elevated levels of DNA damage as well as decreased Ser/ Thr kinase ATM and NBS1 protein activity after double strand break (DSB) induction by intron-encoded endonuclease (I-PpoI), which indicates significant involvement of SIRT1 in the first steps of DSB detection. SIRT1 also deacetylates histone deacetylase 1 (HDAC1), supporting its neuroprotective effect through the nonhomologous end-joining (NHEJ) signaling pathway [145]. The Tat protein induces DSB of the DNA strand, leading to apoptosis if the damage is not repaired [138]. The data above show the potential neuroprotective role of SIRT1 also in the course of HIV neuroinfection.

Another factor in neuroAIDS pathogenesis is mitochondrial toxicity. Postmortem brain specimens from HIV+ patients with documented HAND have been shown to alter mitochondrial functions in neurons and astroglia associated with reduced PGC-1α, the main factor affecting mitochondrial biogenesis by enhancing TFAM transcription, participating in mtDNA replication and transcription. In the tested samples, it was shown that the expression of TFAM and PGC-1α was reduced by 40% in NCI samples and by 50% in HAND samples, compared to non-HAND samples [146]. Research shows that SIRT1 is a factor in promoting mitochondrial biogenesis, activating acetyl-CoA synthetase 2 (AceCS2), then AMPK, which in turn phosphorylates and activates PGC-1α [147]. It has been shown that the Tat protein induces an increase in the potential of the mitochondrial membrane, causing changes in synaptic activity [148]. Mouse primary microglial cells (mPMs) exposed to viral Tat protein showed elevated expression of senescence markers, p21 and p16 proteins; augmented cell-cycle arrest; decreased telomerase activity, increased release of pro-inflammatory cytokines; and down-regulation of SIRT3. The authors indicated that the reduction in SIRT3 activity was mediated by miR-505 and up-regulated by HIV Tat. The authors also demonstrated decreased expression levels of SIRT3 in the striatum of HIV-1 transgenic rats compared to WT rats in an in vivo study, as well as decreased expression levels of antioxidant enzymes, Gpx, SOD2 and CAT, in the prefrontal cortex and in the striatum of HIV Tg rats. The expression of p16, p21 and IL1β were also increased in the samples of frontal cortical brain HIV-infected patients compared to age-matched controls. These studies indicate a protective role of SIRT3 in senescence of microglia as a result of the activity of the Tat protein [149]. Changes in mitochondrial morphology (elongated and broken combs) have been noticed in studies on mice exposed to gp120. The tested animals showed neuropathological changes analogous to HAND. The potential mechanism of this type of change is increased mitofusin 1 (MFN1) and OPA1, which was confirmed by the authors in an in vitro model for rat cortical neurons and SH-SY5Y rat neuroblastoma cells exposed to gp120 [150]. Analogous disturbances in the functioning of the mitochondria are also present in the course of Alzheimer’s disease (AD). SIRT3 in AD has been shown to prevent OS induction through deacetylation of SOD2; disruption of mitochondrial membrane potential; and activation of dynamin-related protein 1 (DRP1) and mitochondrial fission 1 protein (FIS1) factors promoting excessive mitochondrial cleavage, leading to neuronal death due to disrupted ability of mitochondria movement to the synapse and disrupted ATP supply [151]. In addition, SIRT3 prevents the excessive division of mitochondria through deacetylation and activation of OPA1 and MFN1, eliminating the excessive activity of DRP and FIS1 [152]. The viral Tat protein causes neuroinflammation and microglia activation also by promoting mitochondrial dysfunction and disturbing mitophagic processes. In mouse primary microglial cells exposed to HIV-1 Tat, a significantly increased expression of mitochondrial kinase PINK1 was shown. Similar results were obtained in vivo in the frontal cortex of HIV-1 Tg rats through a significantly higher expression of PINK1 being observed compared to WT animals [153]. As research shows, SIRT3, mediated by FOXO3, regulates the expression of genes responsible for the normal processes of mitochondrial fusion and decay (MF2, MRP1, FIS1, TFAM, PCG1α), regulating adequate mitochondrial quantity and quality [151].

Another mechanism of HAND and accelerated aging of infected patients seems to be the activation of astrocytes. It has been shown that miRNA-34a and -138 in the brains of HIV-infected rats were up-regulated compared to age-matched WT rats. These differences were not observed in the younger animals. It was also shown that the abovementioned up-regulation of the miRNAs was inversely correlated with the expression of SIRT1. The authors point to the anti-aging role of SIRT1, which counteracts the deleterious effect of Tat protein, causing up-regulation of glial fibrillar acid protein (GFAP) in a NF-kB-dependent manner, which in turn causes astrogliosis [154].

A relationship with occludin has also been demonstrated. Occludin is one of the BBB proteins in pericytes. It affects the expression of SIRT1 and is a factor that limits HIV transcription. Primary human brain capillary pericytes infected with HIV showed an inverse correlation of HIV replication with occludin expression levels. Both HIV infection and depletion of occludin in the tested cells decreased SIRT1 expression and increased NFκB-p65 acetylation. The authors concluded that occludin, via NFκB/SIRT-1 pathway, modulates HIV-1 transcription, pointing to a significant role of SIRT1 in the pathogenesis of neurological disorders in HIV-infected patients [155]. Chronic activation of miR-142 is also the cause of neuropathology in HAND. In primary human neuron culture and neuroblastoma cells expressing miR-142, decreased monoamine oxidase A (MAO-A) expression was observed compared to controls not expressing miR-142, which is one of the causes of neurotransmitter imbalance and neuronal dysfunction related to HAND. As indicated by the authors, the down-regulation of the neurotransmitter-metabolizing enzyme, MAO-A, is associated with SIRT1-dependent action and the loss of its protective functions as a result of miR-142 [156].

**Figure 7:**
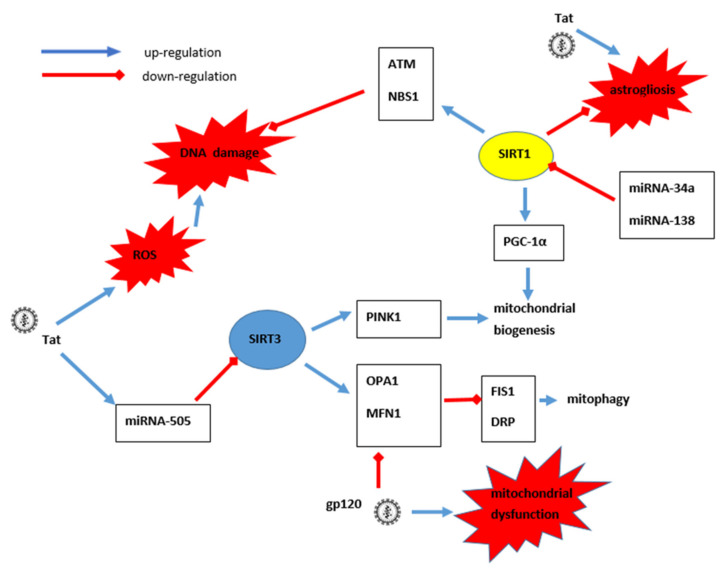
Scheme of connection between sirtuins, neurocognitive disturbances and HIV infection. The Tat protein induces DSB of the DNA strand, leading to OS and apoptosis if the damage is not repaired. SIRT1 activates Ser/ Thr kinase ATM and NBS1 after DSB induction, stabilizing the genome in neurons. SIRT1 is a factor promoting mitochondrial biogenesis through activating PGC-1α. SIRT1 also counteracts the deleterious effect of Tat protein, causing up-regulation of glial fibrillar acid protein in a NF-kB-dependent manner, which in turn causes astrogliosis. SIRT3 prevents disruption of mitochondrial membrane potential and activation of DRP1 and FIS1 factors, promoting excessive mitochondrial cleavage, leading to neuronal death. SIRT3 prevents the excessive division of mitochondria through deacetylation and activation of OPA1 and MFN1, eliminating the excessive activity of DRP and FIS1. SIRT 1, 3, sirtuin 1, 3; PGC-1α, peroxisome proliferator-activated receptor gamma coactivator 1-alpha; PINK1, PTEN-induced kinase 1; DRP-1, dynamin-related protein; OPA1, optic atrophy 1 protein 1; MFN1, mitochondrial fission 1 protein; OS, oxidative stress.

Disturbances in the functioning of dopaminergic neurons (DA) and disturbances in the dopamine mechanism, which are a characteristic feature of Parkinson’s disease (PD), have also been demonstrated in the course of HIV infection [157]. The study of the substantia nigra of HIV-infected individuals showed the expression of neural α-synuclein and β-amyloid deposits, which were not noticed in age-matched control samples [158]. As research shows, the level of SIRT2, increasing with age, is associated with accumulation of a decreased amount of acetylated α-synuclein, and, as a result, increased neurotoxicity and loss of DA neurons. As shown in the PD model, inhibition of SIRT2 by adenylate kinase 1 (AK1) reduced α-synuclein toxicity by reducing its aggregation [159]. As shown by research on mouse hippocampal neurons, by deacetylation of reticulon 4B protein (RTN4B), SIRT2 supports its ubiquitination and disintegration, which in turn inhibits the activity of β-secretase 1 (BACE1) and inhibits the formation of β-amyloid [160]. Figure 7 schematically shows sirtuin’s participation in the developlemt of neurodegenerative diseases in HIV-infected patients.

## 10. Other Disturbances

During the course of HIV infection, different oral disorders occur. It is estimated that HIV-associated oral abnormalities occur in 30–80% of the HIV patient population. Moreover, oral lesions are indicated as early signs of HIV infection and can predict its progression to AIDS [161,162]. It is mentioned that for persons living with HIV who have not yet implemented therapy, the presence of oral manifestations may predict AIDS progression [163]. Furthermore, the presence of oral manifestations in HAART treated patients can be a surrogate marker for HAART efficacy [164]. Although the prevalence of oral lesions like hairy leukoplakia, candidiasis or Kaposi‘s sarcoma has been proven to be lower among patients on HAART [165], other conditions such as oral wartsand salivary gland disease [166,167,168] are more prevalent in antiretroviral treated population as part of immune reconstitution. It is indicated that the junction of tooth and gingiva provides a potentially weak barrier for virulence factors of bacteria. Oral lesions are associated with HIV infection, as well as diverse medication used in the management of patients with HIV disease (e.g., didanosine, indinavir, zidovudine, indinavir). Lately it has been indicated that the introduction of HAART appears to have reduced the incidence of HIV-associated oral lesions [169,170]. There is only little literature to date concerning the role and participation of sirtuins in the development of dental diseases in HIV-infected patients. As shown by Jang et al. [171] in human dental pulp cells (HDPCs) isolated from freshly extracted molar teeth, examining sirtuin gene expression in odontoblastic differentiation (reflected by osteocalcin (OCN) and dentin sialophosphoprotein (DSPP) expression) revealed that all seven sirtuin genes (SIRT1-7 genes) were expressed, and especially the expression of SIRT4 was increased in a time-dependent manner. The authors suggested that SIRT4 could influence the odontoblast differentiation process; however, further research is needed to determine the potential effects of other sirtuins on the odontogenic potential of HDPCs [171]. Kim et al. [172] showed that overexpression of SIRT1 increased mineralized nodule formation, and up-regulated the levels of odontoblastic markers (OCN, ALP, dentin matrix protein-1 and DSPP) as well as angiogenic markers (fibroblast growth factor-2, vascular endothelial cadherin and platelet endothelial cell adhesion molecule 1), while SIRT1 inhibition of SIRT1 down-regulated the expression of those genes. Down-regulation of the SIRT6 decreased the mineralization effect in HDPCs, and overexpression of SIRT6 increased mineralization effects, such as calcium nodule formation, ALP activity and odontoblast differentiation marker genes [172]. Some literature data indicated the role of SIRT1 in the regulation of precancerous oral lesions and oral cancer. However, its biological role in the regulation of oral cancer is not yet fully understood [173]. This seems to be important in light of increased development of such disorders in HIV-infected patients and due to the fact that the biological role of sirtuins in cancer is constantly being recognized and growing [8,10]. SIRT1 has been widely studied and yet there are conflicting results regarding the association between the two, as SIRT1 is known to suppress or promote cancer depending on its cellular content or type [173,174]. The expression level of SIRT1 has been shown to play an important role in the pathogenesis of oral squamous cell carcinoma (OSCC). A statistically significantly lower expression of SIRT1 in resected specimens from patients with OSCC has been demonstrated compared to the control group [175]. The influence of SIRT1 on epithelial e-cadherin activity is indicated as a potential mechanism, which allows the preservation of the integrity of the epithelium and preventing of metastases in oral cancer. SIRT1 also prevents further neoplastic transformation in fibroblasts through its suppressive effect on TGF-β [173].

## 11. Conclusions

The introduction of HAART has significantly increased the effectiveness of HIV treatment, as demonstrated by statistical data on the decreasing mortality from AIDS-defining diseases. Currently, the challenge is to develop appropriate procedures, therapeutic schemes and preventive measures to reduce the risk of or delay the occurrence of non-HIV associated conditions. Therefore, it is important to know the detailed mechanisms in the development of the abovementioned diseases. Data discussed in this review indicate the potential involvement of sirtuins in cellular signaling pathways shared with some viral proteins, leading to osteoporosis (SIRT1, SIRT6), T2DM and insulin resistance (SIRT1, SIRT2, SIRT3, SIRT6), liver diseases and lipid metabolism disorders (SIRT1, SIRT3, SIRT6), cardiovascular diseases (SIRT1, SIRT2, SIRT4, SIRT5, SIRT6), kidney diseases (SIRT1, SIRT3), neurocognitive disorders (SIRT1, SIRT2, SIRT3) and oral diseases (SIRT1, SIRT4). Therefore, it seems that further examination of sirtuin expression in HIV-positive patients and possible regulations of their action to avoid comorbidities are required.
